# Correction to ‘A scalable CRISPR-Cas9 gene editing system facilitates CRISPR screens in the malaria parasite *Plasmodium berghei*’

**DOI:** 10.1093/nar/gkaf757

**Published:** 2025-07-30

This is a correction to: Thorey K Jonsdottir, Martina S Paoletta, Takahiro Ishizaki, Sophia Hernandez, Maria Ivanova, Alicia Herrera Curbelo, Paulina A Saiki, Martin Selinger, Debojyoti Das, Johan Henriksson, Ellen S C Bushell, A scalable CRISPR-Cas9 gene editing system facilitates CRISPR screens in the malaria parasite Plasmodium berghei, Nucleic Acids Research, Volume 53, Issue 2, 27 January 2025, gkaf005, https://doi.org/10.1093/nar/gkaf005

The authors have identified an issue with the PbHiT vector online design tool. Specifically, when the gRNA is located on the reverse strand, the tool incorrectly designed the corresponding homology arms. This error has now been corrected.

To address the issue, the authors have:

- Issued a warning message on the PbHiT design web tool: https://pbhit-crispr-design.serve.scilifelab.se/search
- Updated and deposited the corrected code on Zenodo (https://zenodo.org/records/14000341) and GitHub (https://github.com/srchernandez/PbHiT-CRISPR-Bushell-Lab).

The revised code resolves the design flaw and has been implemented in the web tool.

Importantly, this issue does not affect the findings reported in the article, as all vectors used in the study were designed prior to the development of the automated, whole-genome vector design feature—where the error was introduced.

However, it does impact Supplementary Table S4, which was generated using sequences from the automated tool. This table has now been corrected, and the published article has been updated accordingly.

