# Supplementary material for: A scalable CRISPR-Cas9 gene editing system facilitates CRISPR screens in the malaria parasite Plasmodium berghei
Source: Nucleic Acids Res. 2025 Jan 22;53(2):gkaf005. doi: 10.1093/nar/gkaf005 (PMC11754126; doi:10.1093/nar/gkaf005)
Supplement: gkaf005_Supplemental_File [file gkaf005_supplemental_file.pdf]

Fig. S1

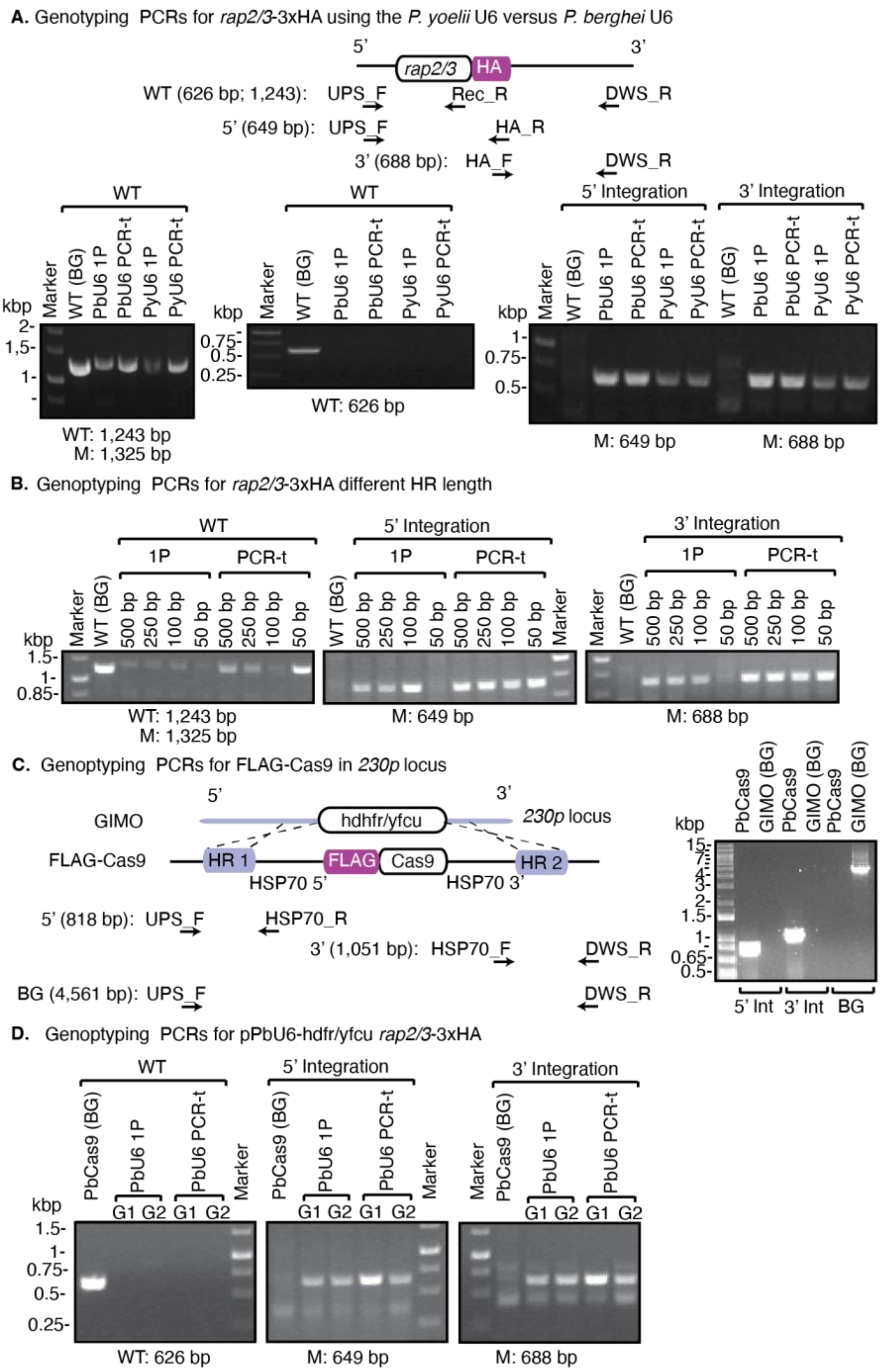

Figure S1: Genotyping PCRs for Figure 1. (**A**) Genotyping PCR for *rap2/3*-3xHA using either *P. yoelii* U6 or *P. berghei* U6 promoter for one-plasmid (1P) and PCR-template (PCR-t) approaches. The schematic diagram shows the location of relevant primers to confirm correct integration and presence of WT DNA. The WT serves as background (BG) line control. M = mutant (*rap2/3*-3xHA here). (**B**) Genotyping PCRs for *rap2/3*-3xHA with different homology region (HR) length. Primers are shown in panel A. (**C**) Genotyping PCRs for the integration of FLAG-Cas9 (PbCas9) into the *230p* locus. The schematic shows where primers are located. (**D**) Genotyping PCR for *rap2/3*-3xHA using the pPbU6-hdhfr/yfcu plasmid transfected into the FLAG-Cas9 (PbCas9) motherline. Schematic diagram in panel A displays where primers are located. G1 = gRNA 1 and G2 = gRNA 2. All primer sequences are found in **Table S1**. Full-length agarose gels are shown in **Fig. S2**.

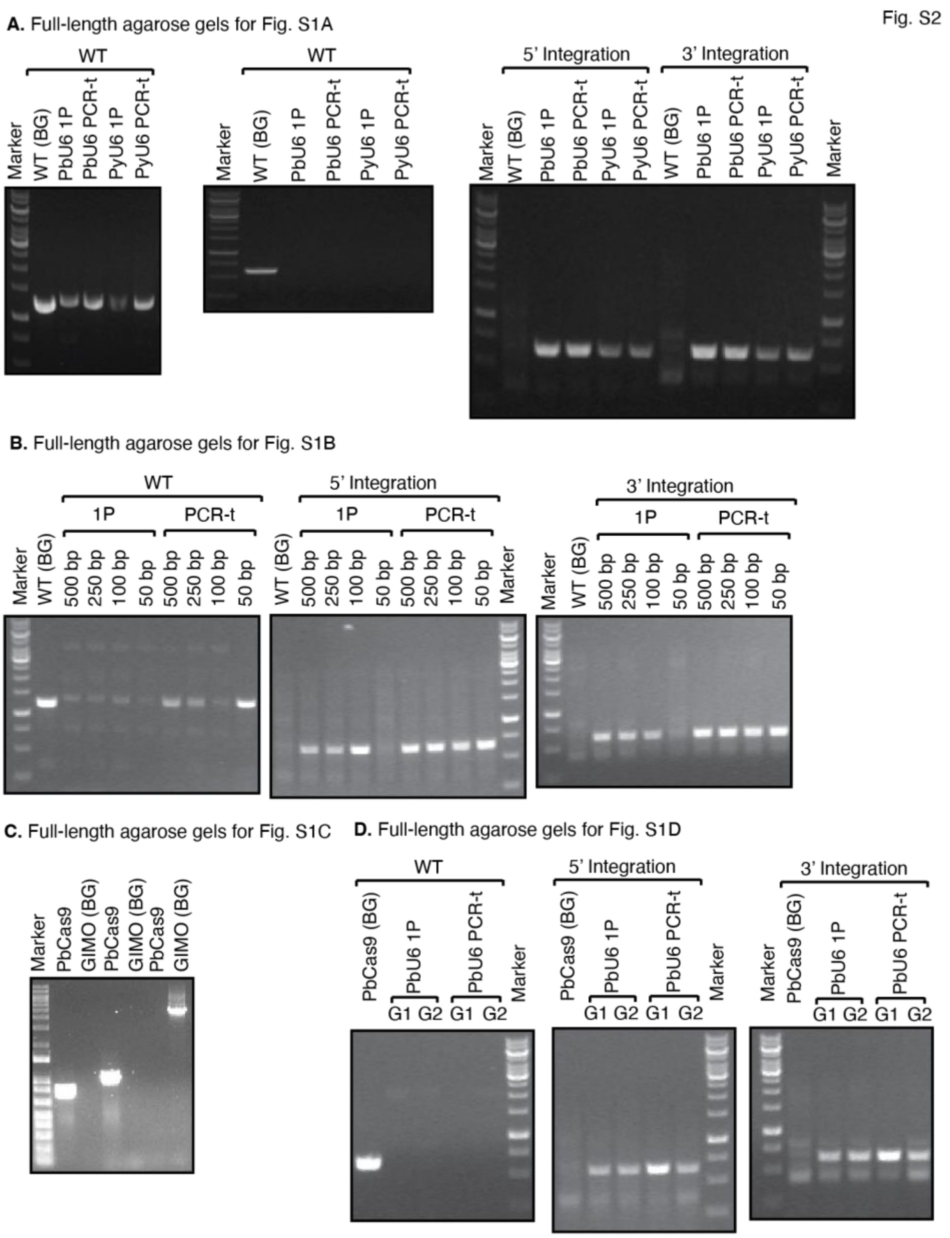

Figure S2: Full-length agarose gels for Figure S1.

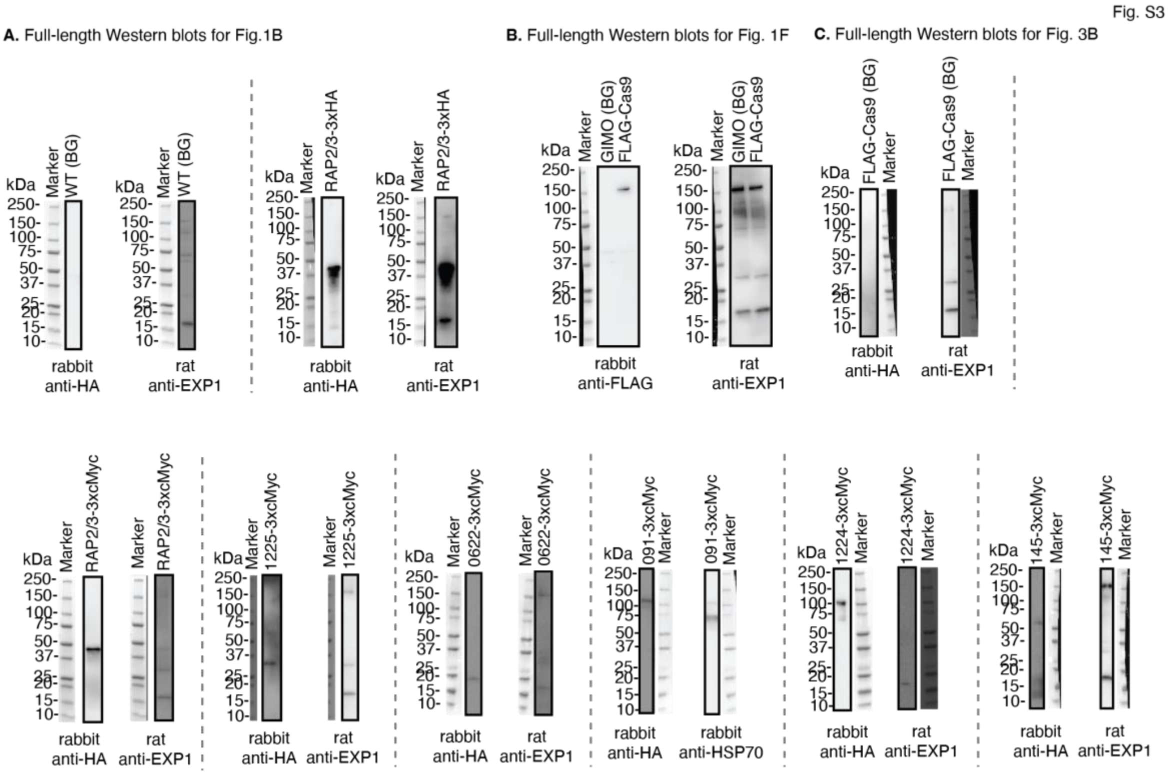

Figure S3: Full-length Western blots for Figures 1 and 3.

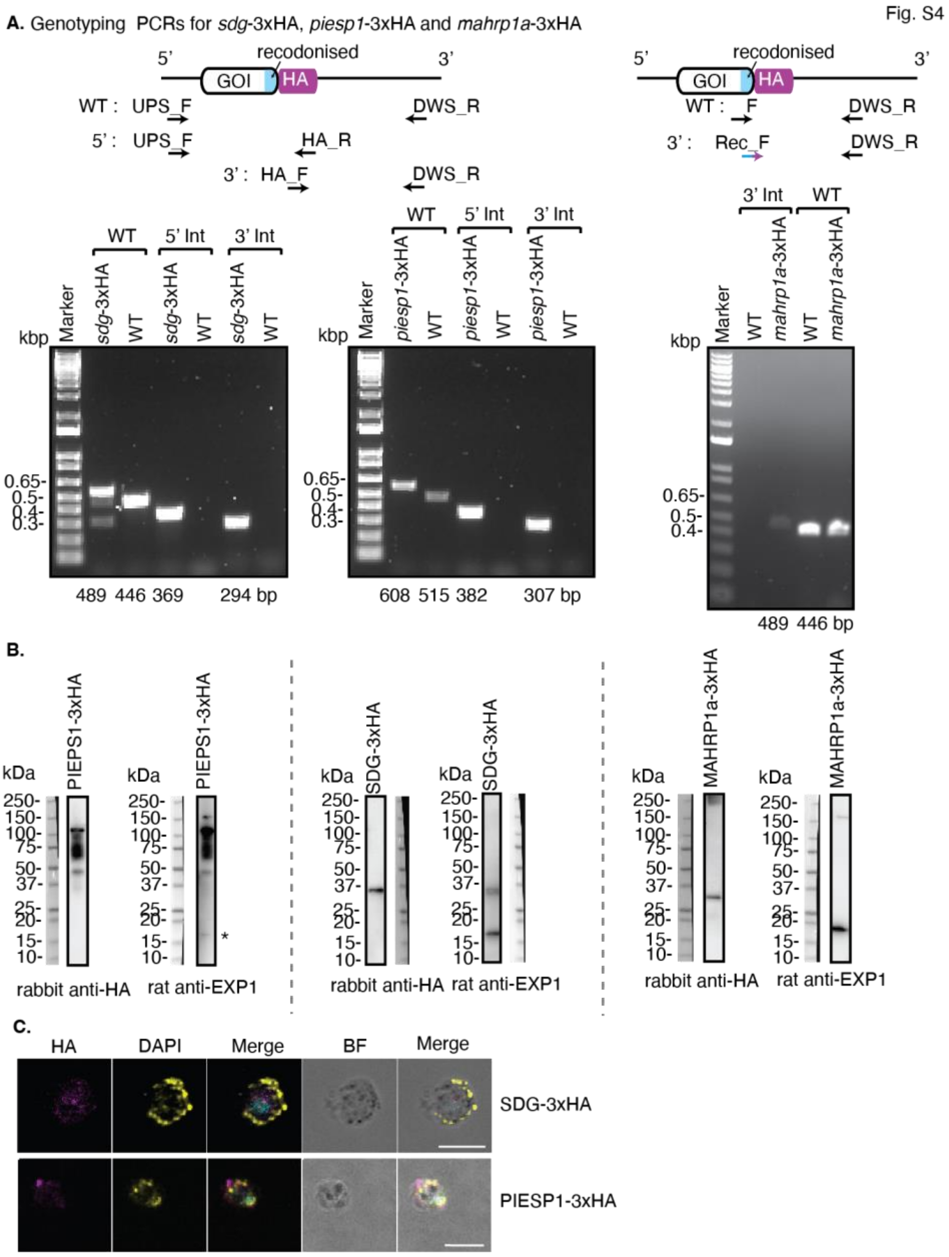

Figure S4: Additional genes tagged using PCR-template approach. (**A**) Genotyping PCRs for *sdg*-3xHA, *piesp1*-3xHA and *mahrp1a*-3xHA lines that were generated using PCR-template approach. Schematic diagram shows primer localisation, one for *sdg* and *piesp1* (left) and another one for *mahrp1a* (right). For *mahrp1a*, the 3' integration had a forward primer with the recodonised sequence and part of the HA tag, which is not present in WT parasites. All primer sequences are found in **Table S1**. (**B**) Expression of tagged proteins was observed by Western blot, where anti-HA detects protein of interest and anti-EXP1 serves as a loading control. WT parasites were used as negative control for the tag. (**C**) Immunofluorescence assay was used to localise both SDG-3xHA and PIESP1-3xHA where anti-HA detects proteins and anti-EXP1 is used as a marker for the parasitophorous vacuole membrane. Scale bars = 5 µm.

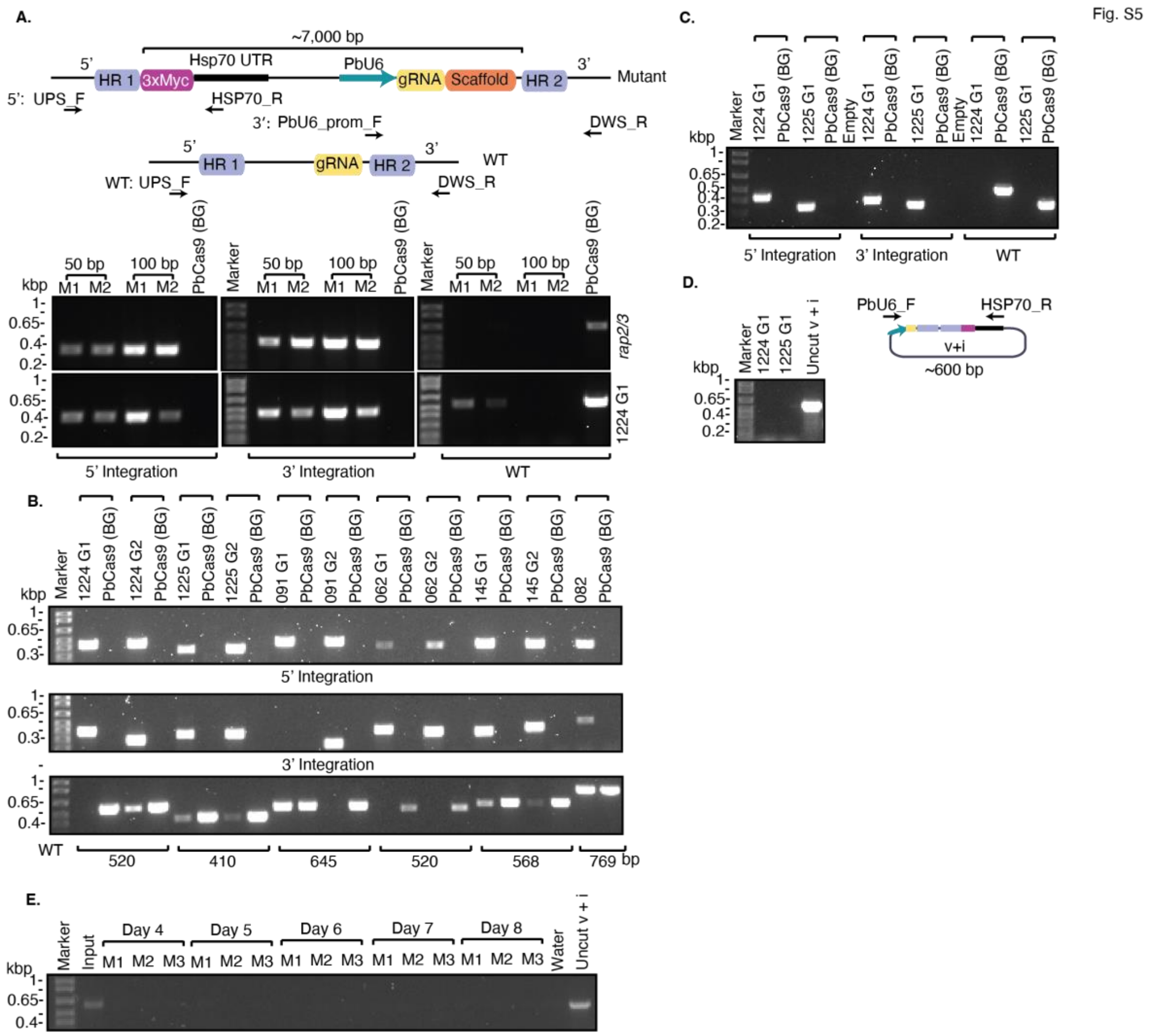

Figure S5: PCRs for Figure 3 and 4. (**A**) Genotyping PCRs for pPbHiT transfections for 1224200-3xcMyc and *rap2/3*-3xcMyc comparing 50 bp and 100 bp HR length. Two independent transfections are shown, mouse 1 (M1) and mouse 2 (M2). PbCas9 was used as background (BG) control for the WT locus. Schematic above shows primer localisation for integration and WT PCRs. M = mutant. (**B**) Genotyping PCRs for the pHiT tagged lines using 100 bp HR length. Primers are shown in the schematic in Panel A. (**C**) Genotyping PCRs for the second transfection of pPbHiT 1224200-3xcMyc and 1225600-3xcMyc where DNA was not gel extracted post *Avr*II digest. Schematic in panel **A** shows primer localisation. (**D**) No episomal plasmid was detected in genomic DNA of transfections where DNA was not gel extracted post *Avr*II digest, where pPbHiT + insert (i) serves as positive control for the presence of an episomal plasmid. (**E**) No episomal plasmid was visible in 22X knockout vector pool on collection days (day four to day eight). All primer sequences are found in **Table S1**.

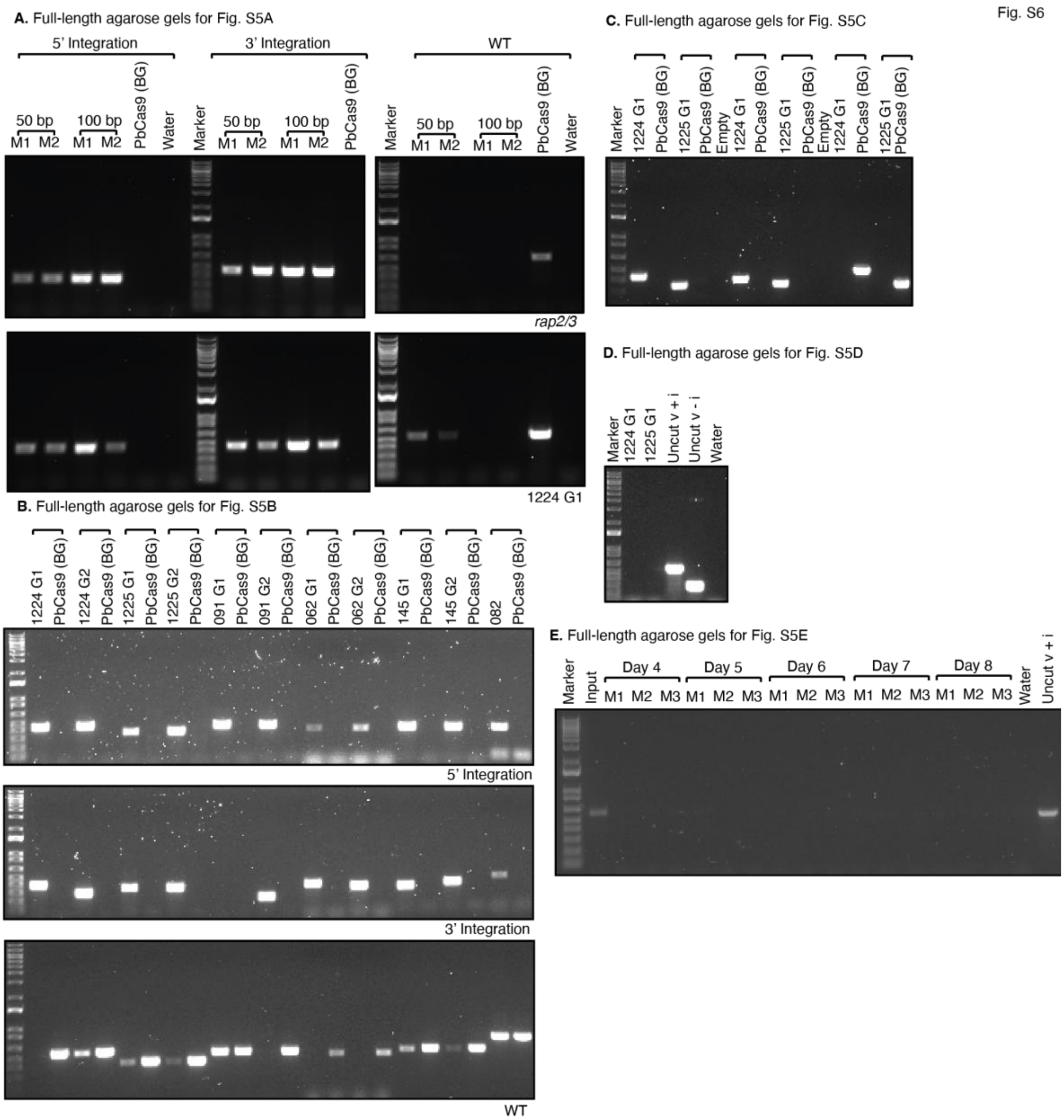

Figure S6: Full-length agarose gels for Figure S5.

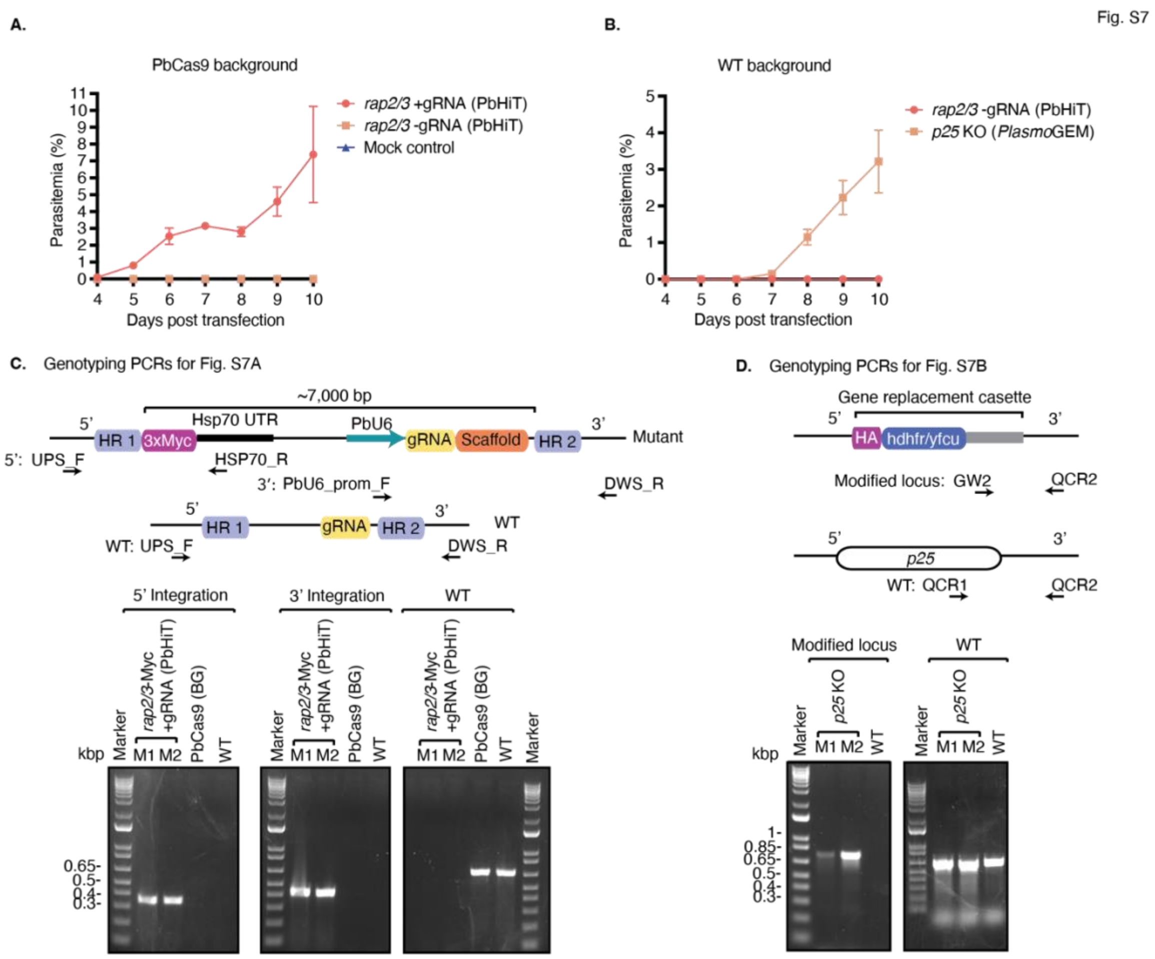

Figure S7: Control experiments confirm that both the Cas9 nuclease and gRNA are needed for successful editing of *P. berghei* using the PbHiT approach.

(**A**) To confirm that both Cas9 and gRNA are needed for the successful transfection of linearised pPbHiT vector with 100 bp homology arms, a 3x-cMyc tagging vector targeting *rap2/3* with (+) or without (-) gRNA was transfected into the PbCas9 motherline, where only the vector containing the gRNA resulted in successful editing. Mock transfections (no DNA) yielded no detectable parasites, demonstrating the lack of detectable spontaneously pyrimethamine-resistant wildtype parasites. (**B**) To further confirm that Cas9 is needed for successful integration of the linearised pPbHiT vector, the *rap2/3* pPbHiT tagging vector with 100 bp homology arms and gRNA (+) was transfected into wildtype schizonts, which resulted in no detectable parasites during the experimental timeframe. A positive control was used to confirm that the schizonts used were viable, where the transfection of the *p25* KO *Plasmo*GEM vector PbGEM-15561 with 2.5 and 5.8 kb homology arms (targeting the blood-stage dispensable *p25* gene) resulted in successful generation of parasite mutants. (**C**) Genotyping PCRs confirm successful integration of *rap2/3* locus with 3xcMyc when transfected with gRNA (+). PbCas9 was used as background (BG) control for the WT locus. (**D**) Genotyping PCRs confirm successful detection of the *Plasmo*GEM knockout vector targeting the *p25* gene (PBANKA_0515000), where *P. berghei* WT is used as a control. The long homology arms of the *Plasmo*GEM vectors prevent integration PCRs, however, PlasmoGEM vectors are not maintained episomally and the vector is only detected upon integration into the genome (8). Two independent transfections are shown, mouse 1 (M1) and mouse 2 (M2). All parasitemia counts can be found in **Table S5** and primer sequences in **Table S1**.

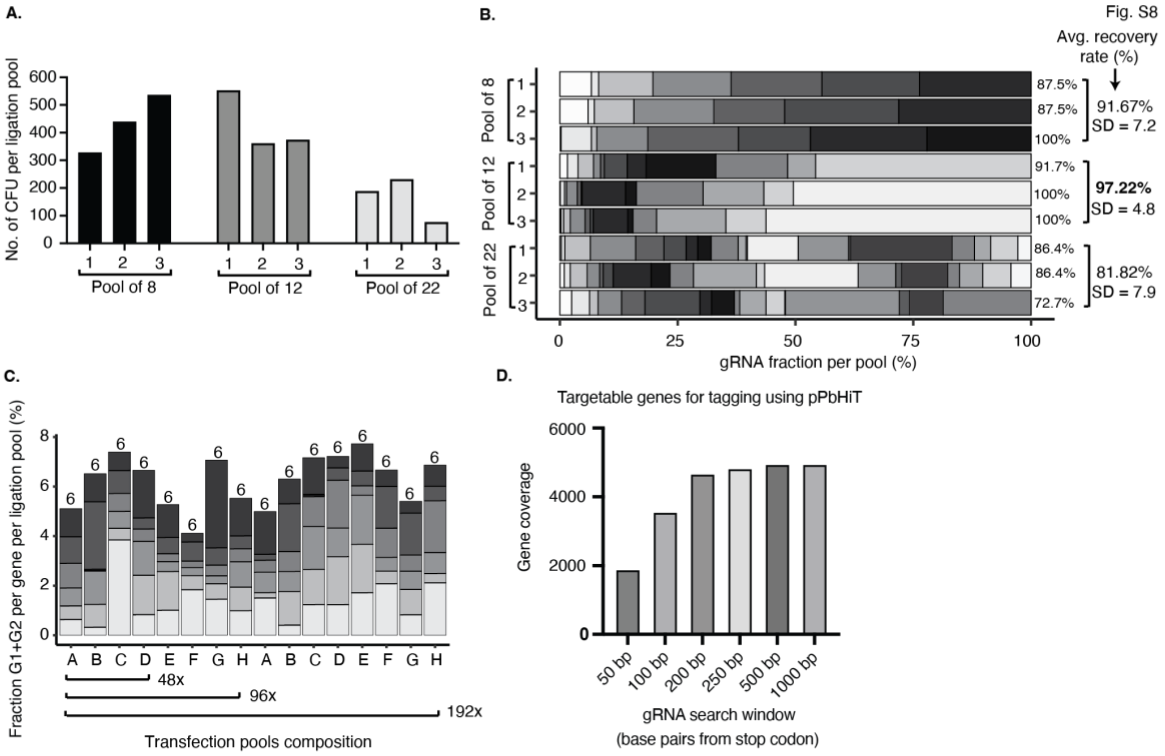

Figure S8: Pooled pPbHit vector ligations. (**A**) Colony numbers and (**B**) NGS results for testing ligation pools of 8, 12 or 22 inserts (synthetic fragments) cloned into pPbHiT in three biological replicates. Recovery rate is given as an average percentage (across replicates) of unique gRNA detected by NGS compared to possible maximum and the absolute number of gRNA for each ligation pool. The percentage of each gRNA within each ligation pool is plotted along the X-axis. SD = standard deviation. (**C**) Vector pool composition used for larger PbHiT CRISPR screen (pools of 48X, 96X and 192X). The proportion of each gene is displayed by combining the two gRNAs targeting the same gene and is plotted along the Y-axis. This means one row (12X) on a 96-well plate should have six stacks in the histogram. (**D**) Coverage of *P. berghei* genes targetable for C-terminal tagging when allowing for different maximal distances between stop codon and gRNA.

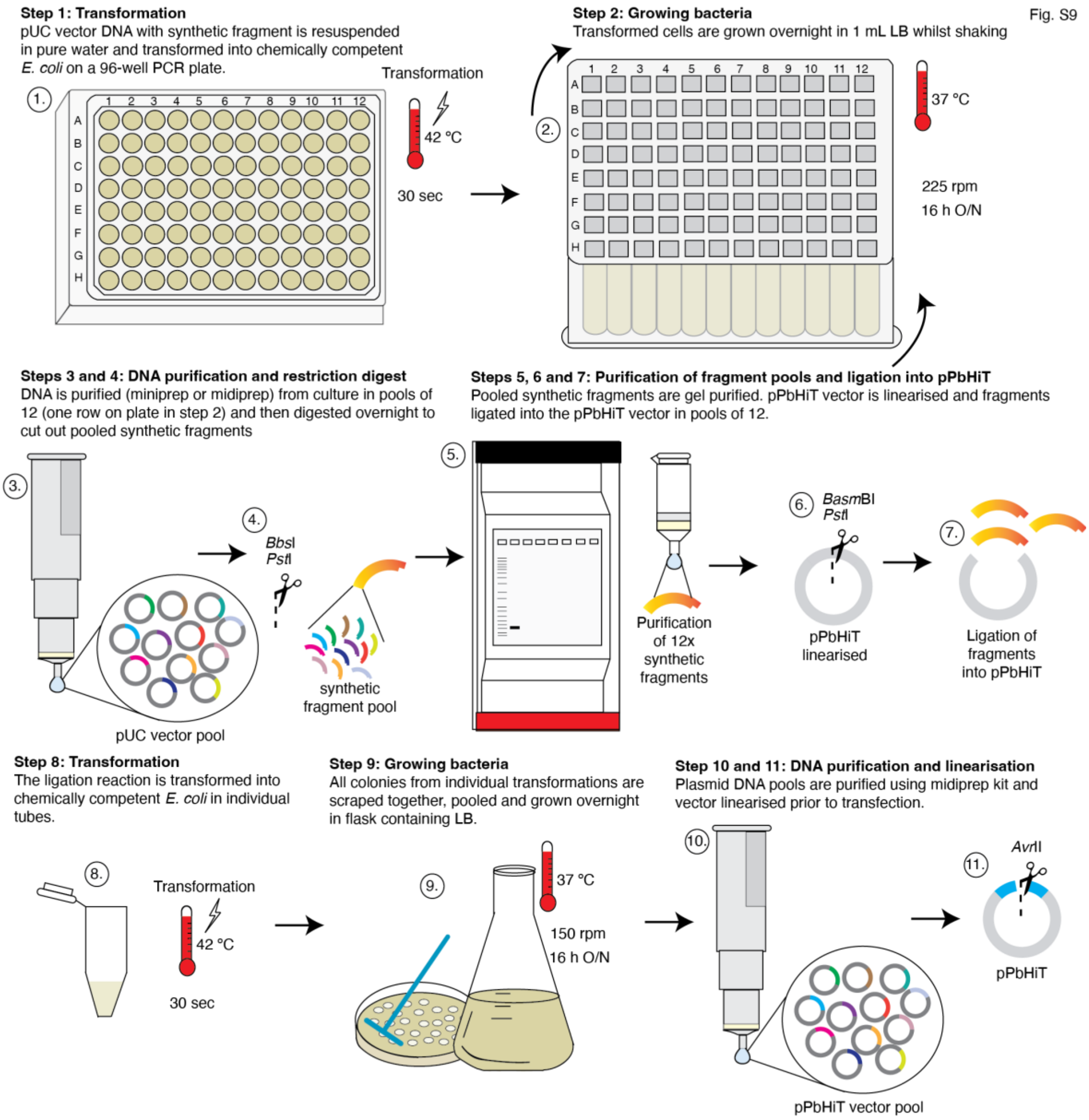

Figure S9: Pooled pPbHiT vector ligations. Experimental workflow for generation of pPbHiT vector pools for CRISPR screens.

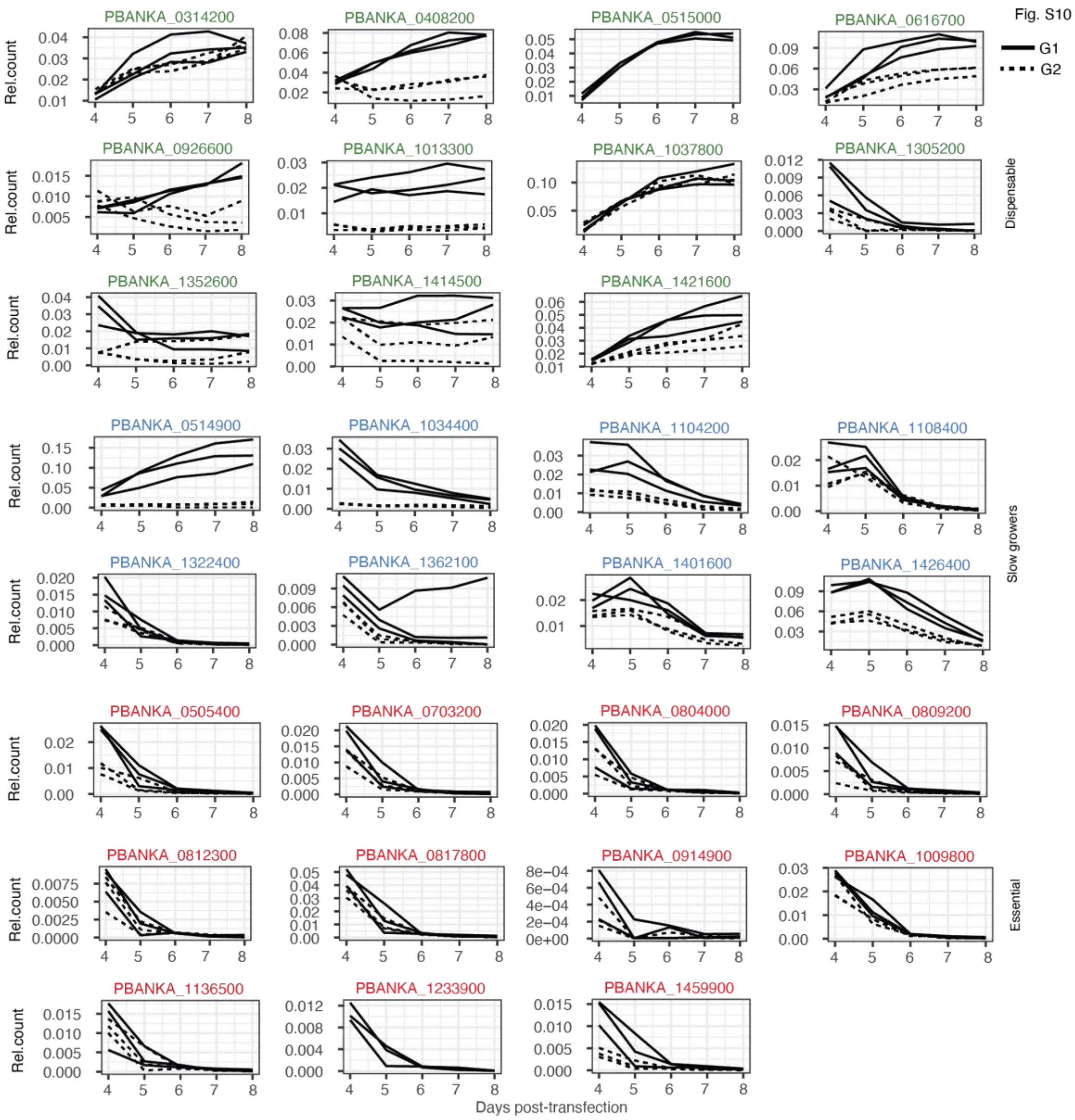

Figure S10: Relative gRNA abundances for all genes in the 48X CRISPR pool. The relative abundance of mutant gRNA (barcodes) was calculated for each sample as the proportion of the sum counts for all predicted dispensable genes present in the pool. Each line represents the change in relative abundance within one single mouse where the two gRNAs targeting the same gene are plotted separately. PBANKA IDs are coloured based on previously published blood stage growth phenotypes (9): dispensable (green), slow growers (blue) or essential (red). G1 = guide one, and G2 = guide two.

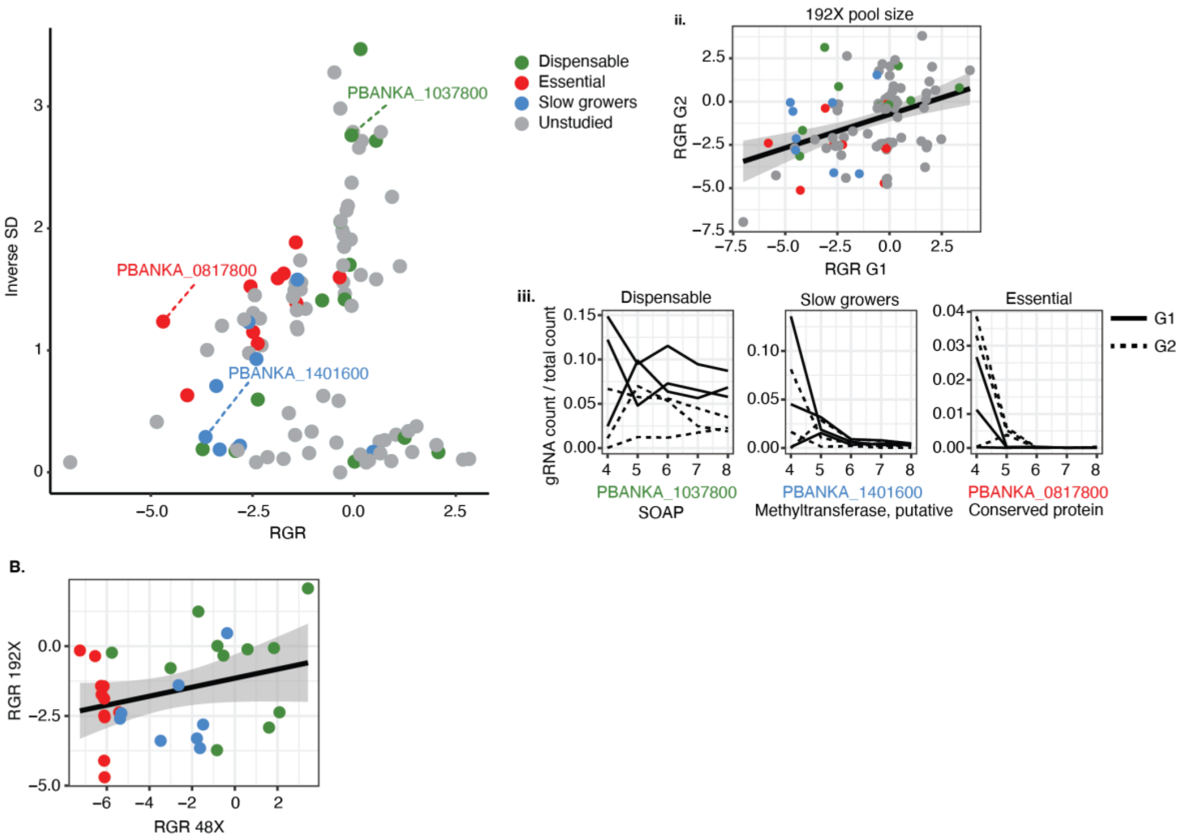

Figure S11: PbHiT CRISPR screen using a pool of 192X vectors. **(A)** Analysis of 192X gRNA CRISPR screen targeting 96 genes, including 24 target genes with known blood stage growth phenotypes and overlapping with the 48X gRNA pool. (**i**) Scatter plot of mutant relative growth rates (RGR) plotted against the inverse of standard deviation (1/SD). (**ii**) Correlation of RGR between two gRNA targeting the same gene. (**iii**) Selected line graphs of mutant relative abundances with each line representing counts from a single gRNA in an individual mouse. The R^2 values for the RGR correlation between gRNA1 and gRNA2 in the 192X vector pool was 0.15. (**B**) Correlation between mutant RGR for genes overlapping between the 48X and 192X pool (R^2 = 0.09). Genes are coloured based on published blood stage growth phenotypes (9): dispensable (green), slow growers (blue) or essential (red). Genes lacking *Plasmo*GEM knockout screen phenotype are classified as unstudied (grey).

Table S1: Primer sequences and fragment sequences used in this study

Purpose	Primer/fragment name	Sequence (5’-->3’)	Description
Cloning of spCas9 GIMO vector	Cas9_GIMO_F_Gib	CAATTACAATTAAAAAAAGGATGGACTATAAGGACCACGAC	Cloning of spCas9 into pL1694, primer with Gibson overhang
	Cas9_GIMO_R_Gib	CAGAACAATAATAGCGGCCTTACTTTTTCTTTTTTGCCTGGCC	Cloning of spCas9 into pL1694, primer with Gibson overhang
Cloning of new CRISPR/Cas9 vector backbones	PbU6_F_Gib	CGCAGCCTGAATGGCGAATGATCGAGAACTATTGTTTCCTTTTTTGTTTTTTTATTTTTAATTAAAATG	Cloning of PbU6 into PyCM or PyCS, primer with Gibson overhang
	PbU6_R_Gib	GCTCTAAAACTGAGACGAGGCCTCTACGCGTCTCCAATAATATTGTATAACTCGAAGTATGC	Cloning of PbU6 into PyCM or PyCS, primer with Gibson overhang
	PbU6_BbsI_R	ACTTGCTATTTCTAGCTCTAAAACAGGTCTTCTCGAAGACCCCAATAATATTGTATAACTCGAAGTATG	Cloning of PbU6 into PyCM or PyCS, primer with Gibson overhang
Building of pPbHiT vector	pPbHiT building synthetic fragment	ATATTATTGGAGACGCTGCAGgaacaaaaacttattagcgaagaagatcttGAACAAAAATTAATAAGTGAAGAAGATTTA GAGCAGAAGTTGATTTCAGAGGAGGACTTGgtcgacGCGGCCGCgTAGATAAATAATTTTTTTTTTTATATTATTGT TCTGTACTTCTTTTGTGAATAGTATTTTTACATATGTACTGTTAATTTTAAAATGTGGAATGCCACGAACATATTTTT TCGAACAAAATATATATTTTTTGTGTCGAAATACATAAATTTGTGTACCTCCTTTTTGTTGTATATATGAAAAAGGAA TAAAGTGAATGGAGTCGAATTACTCTAGACCCTACAACTTGTATATCACTACTTTATAATACTATAATATTATTATTAA CATTAAACTATAGAAGAAATATCAACAATACTTATGTATTTGTTCAAACTTTATCAGTATAGTTTGGTAATCGCATAT CATATATTTGTTATGTGTAATATTTTATGAATATGCGTCTTTTTTTTTAACTATAAATAATTCAAAATAAAATAACATAA TTAACATTTGCAAGCATAATTAAGATGATTACTTTCACATTTCATCCAAAGTGGAAAATGACCACCACATTTGATC GAAGCTTTTACCACTTTTGATGCATTAGCTCTGAAATGTATACAAGCTTTCATTTGTTAATGTTACAAAGGGAAGT TTTTATTTGGGGGCACATTAAAAGTGAAAAAATTAAAAGTTAAAAAAGTTAAAAAATGATCGGTGTAGTTGGTC ATATAGACATGTTGTTATGCAAGTCTTTCAAAACGTTCCTCATAAAATAAAAGGAGGGATCTAGAGAGCTTTCAAT TCTCCAAACGGTATTATTGGTATTTTCTGAAAACTGCGTTTCCTTGTTTTTATTTATATTTGACGTC	Synthetic sequence used to build pPbHiT upon modification of pPbU6-hdhfr/yfcu
Diagnostic primers CRISPR/Cas9 vectors	gRNAseq_R	CAAATAGGGGTTCCGCGCAC	Diagnostic PCR and sequencing of gRNA insertion into pPbU6-hdhfr/yfcu and pPbU6-hdhfr/yfcu-Cas9
	hsp70UTR_R	TATGTTCGTGGCATTCCACAT	Diagnostic PCR and sequencing of gRNA insertion into pPbHiT

	PbU6prom_F	ACATATGCGCATACTTCGAGTTATAC	Diagnostic PCR and sequencing of gRNA insertion into pPbHiT
PBANKA_1101400 (rap2/3) construct (not pHiT)	Recodonized rap2/3 fragment	CCAGGAGACATTTACCTTGACACCAAAATGGTGGGTGCA	
500 bp construct	PB_Rap23_gRNA1_F	TATTGTATTTGGATACTAAGATGGT	Guide RNA oligo
	PB_Rap23_gRNA1_R	AAACACCATCTTAGTATCCAAATAC	Guide RNA oligo
	PB_Rap23_gRNA2_F	TATTGTATCCAAATATATATCACC	Guide RNA oligo
	PB_Rap23_gRNA2_R	AAACGGTGATATATATTTGGATAC	Guide RNA oligo
	PB_Rap23_HA_5HR_F	CTATGACCATGATTACGCCAGAGGTTAATGATAAACGCATAGATGC	Amplification primer
	PB_Rap23_HA_3HR_R	GGACCATGGCATGGGTACCAGAATATCCTAAAAGCATGACTATACC	Amplification primer
250 bp construct	Rap23_5HR_250bp_F	CTATGACCATGATTACGCCACATACGATTTTTGGACAATTTTAGTATAGCAATAC	Amplification primer
	Rap23_3HR_250bp_R	GGACCATGGCATGGGTACCACTAAAAATATGGATATAAAATAAAGGGAATAAAC	Amplification primer
100 bp construct	Rap23_5HR_100bp_F	CTATGACCATGATTACGCCACATATCATCCGAATTAATCTTCTTAAGAGAAAG	Amplification primer
	Rap23_3HR_100bp_R	GGACCATGGCATGGGTACCACACGATTTTATCTAAATATTTTTCTAATAG	Amplification primer
50 bp construct	Rap23_5HR_50bp_F	CTATGACCATGATTACGCCAGTTACACCAATAAATATATGAATTTACCAGGAGAC	Amplification primer
	Rap23_5HR_50bp_R	GGACCATGGCATGGGTACCACTATTACATAAAAAATAAAAACTTAATAGCTC	Amplification primer
PBANKA_1101400 (rap2/3) genotyping	Rap2/3_UPS_FW	GCAAAGTTTATAGATGATCCTATG	Genotyping primer
	Rap2/3_DWS_RV	GTATATTTATATGTGTATTCCCC	Genotyping primer
	Rec_R	CTTAGTATCCAAATATATATCACC	Genotyping primer
	HA_F	CGCTTATCCCTACGATGTGCCC	Genotyping primer
	HA_R	CATCATATGGATATGCACCCACC	Genotyping primer
Note: Rap2/3 HR actual sizes	**5' HR (lenght referred in text)**	**Actual lenght**	
	50 bp	65 bp	
	100 bp	130 bp	

	250 bp	268 bp	
	500 bp	582 bp	
	**3' HR (lenght referred in text)**	**Actual lenght**	
	50 bp	50 bp	
	100 bp	126 bp	
	250 bp	281 bp	
	500 bp	545 bp	
PBANKA_0522400 (sdg) PCR-template approach	SDG_sgRNA1_F	TATTGACAATACTGAATAGCCATTT	Guide RNA oligo
	SDG_sgRNA1_R	AAACAAATGGCTATTCAGTATTGTC	Guide RNA oligo
	SDG_sgRNA2_F	TATTGACTTAAGATAGTCAGACAAA	Guide RNA oligo
	SDG_sgRNA2_R	AAACTTTGTCTGACTATCTTAAGTC	Guide RNA oligo
	SDG_HR1_F	CTGAATGTATCATCAGAAAAATGAAG	Amplification primer
	SDG_HR2_R	GTAAAGTCTAAAGGATAAATATGAATATCC	Amplification primer
PBANKA_0522400 (sdg) genotyping	SDG_UPS_FW	CAAGTTCAGTGTCATACCCCTATGT	Genotyping primer
	SDG_DWS_RV	CTGATTACAAAGGTGCATTTATATG	Genotyping primers
	HA_F	TACCCATATGACGTTCCAGACTACGCGT	Genotyping primer
	HA_R	AGCGTAGTCAGGTACGTCGTAAGGGTAA	Genotyping primers
PBANKA_0408500 (piesp1) PCR-template approach	PIESP1_sgRNA1_F	TATTGTTTCGGCTCTCTCATTATAT	Guide RNA oligo
	PIESP1_sgRNA1_R	AAACATATAATGAGAGAGCCGAAAC	Guide RNA oligo
	PIESP1_sgRNA2_F	TATTGAAATATTCATCTGGAGTGT	Guide RNA oligo
	PIESP1_sgRNA2_R	AAACACACTCCAGATGAATATTTC	Guide RNA oligo
	PIESP1_HR1_F	TCTGAAGAAGATGATGAAAATGAT	Amplification primer
	PIESP1_HR2_R	GTATATCAAATGAGCATGTACATGA	Amplification primer

PBANKA_0408500 (piesp1) genotyping	PIESP1_UPS_FW	CGTAAATAATTACGTTGTAGAAAATACTG	Genotyping primer
	PIESP1_DWS_RV	CGAATAGAGCTTATTTTTGTTTAC	Genotyping primers
	HA_F	TACCCATATGACGTTCCAGACTACGCGT	Genotyping primer
	HA_R	AGCGTAGTCAGGTACGTCGTAAGGGTAA	Genotyping primers
PBANKA_1145800 (mahrp1a) PCR-template approach	MAHRP1a_sgRNA1_FW	TATTGAGACCCCCAGGATGTACTTG	Guide RNA oligo
	MAHRP1a_sgRNA1_RV	AAACCAAGTACATCCTGGGGGTCT	Guide RNA oligo
	MAHRP1a_sgRNA2_FW	TATTGAAAGCTCATGGTACTACTAG	Guide RNA oligo
	MAHRP1a_sgRNA2_RV	AAACCTAGTAGTACCATGAGCTTT	Guide RNA oligo
	MAHRP1a_HR1_FW	GTCTGTGCATGTGTTAAGTGC	Amplification primer
	MAHRP1a_HR2_RV	CATGTACATCCATTAGAGCTTGAT	Amplification primer
PBANKA_1145800 (mahrp1a) genotyping	MAHRP1a_FW	TGCGGAGGACATAAAGCTC	Genotyping primers
	MAHRP1a_Rec_FW	GACAAATGAATTCTATCCATACGACGT	Genotyping primer
	MAHRP1a_DWS_RV	AAAGTTAAAATTCACCATTTAGCC	Genotyping primers
PbCas9 genotyping	HSP70_UTR_FW	GGAGGGATCTAGAGAGCTTTCAA	Genotyping primers
	HSP70_UTR_RV	ACAAATTCAATGAACTTCTAATATGACTC	Genotyping primers
	p230p_UPS_FW	GAATTGTATATGGTAAAGAACCTACTAACAC	Genotyping primers
	p230p_DWS_RV	GAATAGTGACTTTCAGTGAAATCGCAAA	Genotyping primers
p25 KO genotyping	P25_GW2_RV	CTTTGGTGACAGATACTAC	Genotyping primers
	P25_QCR1_FW	ACCCCATGTTCATTAGTTTGCGGT	Genotyping primers
	P25_QCR2_RV	TGCATATGCACGACTAGCTCA	Genotyping primers
Illumina libraries for gRNA sequencing	BC_pHiT_illumina_F	ACACTCTTTCCCTACACGACGCTCTTCCGATCTCGAATGCACTATTCATTTTATGGGG	Amplifies gRNA from all pPbHiT vectors and parasites, with overhang for priming in second PCR.

			Nested PCR 1 for seqeuncing libraries.
	BC_pHiT_illumina_R	TCGGCATTCCTGCTGAACCGCTCTTCCGATCTGACTCGGTGCCACTTTTTCA	Amplifies gRNA from all pPbHiT vectors and parasites, with overhang for priming in second PCR. Nested PCR 1 for seqeuncing libraries..
	PE 1.0	AATGATACGGCGACCACCGAGATCTACACTCTTTCCCTACACGACGCTCTTCCGATC*T	Standard Illumina library primer with Illumina adaptors to attach to flow cell. Nested PCR 2 for seqeuncing libraries.
	Illumina index primer 1-96	Sequences available from Bushell et al 2017 (PMID: 28708996)	Custom indexed Illumina library primer to allow sample multiplexing, with Illumina adaptors Nested PCR 2 for seqeuncing libraries.
	Illumina custom index seq primer	AAGAGCGGTTCAGCAGGAATGCCGAGACCGATCTC	Custom Illumina index sequencing primer
pHiT 50 bp tagging constructs primers	PB_1224200_g1_50bp_FW	GAAGACggTATTTTAGATATGCATACAAA	
	PB_1224200_g1_50bp_RV	CTGCAGTGACTTGTTTTGATTATGC	
	PB_1101400_g1_50bp_FW	GAAGACggTATTAGAAAATAAGTAAATTTGATG	
	PB_1101400_g1_50bp_RV	CTGCAGTGCGCCGACCATCTTAGTA	
pHiT protection base pairs primers	All_50bp_RE_prot_bp_F	AGTCCATAGAAGACggTATT	
	PB_1224_50b_prot_bp_R	TAATGTTTCTGCAGTGACTTG	
	PB_11014_50b_prot_bp_R	TAATGTTTCTGCAGTGCGCC	
PbHiT 50 and 100 bp genotyping	PB_1224200_UPS_FW	GCCTTGAAACAACTAGGAGATATGAT	Genotyping primers
	PB_1224200_DWS_RV	GGATTGATTGCATAGCAACCATATA	Genotyping primers

	PB_1225600_UPS_FW	GGCTCTATATGAAAAATGCAAAATGAAGG	Genotyping primers
	PB_1225600_DWS_RV	GTTTGGAGTTGCTTATACTTGTAACATG	Genotyping primers
	PB_0914500_UPS_FW	ATGGAAATGTGAAAATTATACCTTCAAAGG	Genotyping primers
	PB_0914500_DWS_RV	TATAATATGCACACAAACAACGTGC	Genotyping primers
	PB_0622900_UPS_FW	CAGAATGGATACCCCTGATCCATT	Genotyping primers
	PB_0622900_DWS_RV	TTCGTAACGTATATTGCGTTTACATTG	Genotyping primers
	PB_1451000_UPS_FW	GACAACATTTGCTGTGCATTTAGTG	Genotyping primers
	PB_1451000_DWS_RV	TCCATTAAGTTGTGCTAATAAACAATTTTC	Genotyping primers
	PB_0829400_UPS_FW	CTCCTCTGAAAATGTAAAAAAATGCCA	Genotyping primers
	PB_0829400_DWS_RV	GTTTCTCCTTGTTTTTTTGTTGATATATGC	Genotyping primers
	PB_1101400_UPS_Fw	GAATTCTGATTACATATCATCCG	Genotyping primers
	PB_1101400_DWS_Rv	GGTATAGTCATGCTTTTAGGATATTC	Genotyping primers

Table S2: Genes tagged using PbHiT and relevant sequences

Target gene ID	Gene name	Guide RNA	Guide sequence (5’-->3’)	HR length (bp)	HR1	HR2
PBANKA_1224200	DnaJ protein, putative	G1	AGATATGCATACAAAACA	100	AGAAGAAGAAGCAGCAGCAGAAGCAGCAGCAGAAGCTGAATCAACCAAAATTGAGAGGGATGAAAAATACAGTGGTCTAAGGCATAATCAAAACAAGTCA	TGTACTTTCGCTTCGAATTAAATTCTATGACTTTTTTCTCTTTTAATTAAAAAGTGCAGCATCTGTAATATATATAACTCCCTTTGACATTCAAATTCCA
				50	TTGAGAGGGATGAAAAATACAGTGGTCTAAGGCATAATCAAAACAAGTCA	TGTACTTTCGCTTCGAATTAAATTCTATGACTTTTTTCTCTTTTAATTAA
		G2	TCTTGGAATTTGAATGTCAA	100	AGAAGAAGAAGCAGCAGCAGAAGCAGCAGCAGAAGCTGAATCAACCAAAATTGAGAGGGATGAAAAATACAGTGGTCTAAGGCATAATCAAAACAAGTCA	CAAATCAATTTAATTCAAATGCTTGATGATTTATAAAAATTTTATTTAAACATAAAATAAAATAGTAACTTCCTATAATTTTAAGTGCCATACACTGTGT
PBANKA_1101400	rhoptry-associated protein 2/3	G1	AGAAAATAAGTAAATTTGAT	100	AAGTTTTTTTGATGCACTTGATAGTACTTTAAATTGTTACACCAATAAATATATGAATTTACCTGGTGATATATATTTGGATACTAAGATGGTCGGCGCA	TGTTTATTCCCTTTATTTTATATCCATATTTTTAGTTGAAAACTTTTTTATATATTTAAAGGTTATACTGACAAATGGTAATATATTTTCCCCTTGCATC
				50	ATATGAATTTACCTGGTGATATATATTTGGATACTAAGATGGTCGGCGCA	TGTTTATTCCCTTTATTTTATATCCATATTTTTAGTTGAAAACTTTTTTA
PBANKA_1225600	alpha/beta hydrolase, putative	G1	CAAATAAAACCACAAAAAAG	100	CTATTGGGTTGCCAATGGAAAACATAACGATGTTGAATTAATTGACAATAAAAAATTCAACGAAAACATCAAGTTTTTCCTAAATTTTTTAAATAATTCA	AACTTTTTTAAATGTGAAAGTTATCAAAAAAATATATATAATTTGCATGTTATTTTATTATTTTTTGTATTCAAAATATATGTTTATGTAATACCTTATT
		G2	AATGAATTTCCCCTTTTTTG	100	CTATTGGGTTGCCAATGGAAAACATAACGATGTTGAATTAATTGACAATAAAAAATTCAACGAAAACATCAAGTTTTTCCTAAATTTTTTAAATAATTCA	TTTTATTTGAACTTTTTTAAATGTGAAAGTTATCAAAAAAATATATATAATTTGCATGTTATTTTATTATTTTTTGTATTCAAAATATATGTTTATGTAA
PBANKA_0914500	conserved Plasmodium protein, unknown function	G1	ATAACAATAATAATAATACA	100	TTATATTGACATATTTTACGATAAAATGAGCGAAATTATGAAAAACAACATGAATTTGGAATTATTTGGAAAAACATTCTTTTTACATTTGGAAAAAAAA	TATTTTTTTAGTTGCAGTATCTATATGATTCTCTATATATTATATCAAATAATTCCTGTGAAGAAATTTATTTCGTAAGTTTCACAAAATTTTCGAGATT
		G2	TACGAAATAAATTTCTTCAC	100	TTATATTGACATATTTTACGATAAAATGAGCGAAATTATGAAAAACAACATGAATTTGGAATTATTTGGAAAAACATTCTTTTTACATTTGGAAAAAAAA	AGTTTCACAAAATTTTCGAGATTTTTAAGTACCCAGACGCCAGGCTGCTTTAATTTGCATTTAATTTATTATTATTATTTTTTTTAATAATTAACTTTAG
PBANKA_0622900	conserved Plasmodium protein, unknown function	G1	TCCATAATACATATAGCAAA	100	AGTAACTGTTTATAATAAACATGGAGAACCATTATATTTTTATATAAATAAAAAGAAAACAAAAAAAAGACTCCAAACTAAGAAAGAAAAAAAAATAAATCT	ATAAAGTTGTTAGTTTATAATACCTGTCAAAAACATTATTTCGTTGATTTTTATATATGCGTGCATTAAGATAGAATTGTTGTAAATGCTACGCTCATGT
		G2	AACGAAATAATGTTTTTGAC	100	AGTAACTGTTTATAATAAACATGGAGAACCATTATATTTTTATATAAATAAAAAGAAAACAAAAAAAAGACTCCAAACTAAGAAAGAAAAAAAAATAAATCT	GATTTTTATATATGCGTGCATTAAGATAGAATTGTTGTAAATGCTACGCTCATGTTTAATAATTTT

						GTTCCAAGAGATTATATATCATATACATTTGAT G
PBANKA_1451000	conserved Plasmodium protein, unknown function	G1	AAAAAGTATGACC CTATAAG	100	TTATACAAGCGATGACAAATTAACATTACTTGTTA ATAATCAAACGAAAAATTATTCCATAAATCAGAA AAAAAAAAAAAAAAAAAAAAAAAATCTGGAAAA	AACCGTATTTAGTCATTAATGTATTACTATATAT TATTAAATACCGCTAATTTTTTTTTTTTTATGTA CATACATGTATAATTGCATTCCGAGCTATTTG
		G2	AAAAAATAAGGTA TTTCAAT	100	TTATACAAGCGATGACAAATTAACATTACTTGTTA ATAATCAAACGAAAAATTATTCCATAAATCAGAA AAAAAAAAAAAAAAAAAAAAAAAATCTGGAAAA	ATTGTCAATATATATTCCCCTTATAGGGTCATA CTTTTTAACCGTATTTAGTCATTAATGTATTAC TATATATTATTAAATACCGCTAATTTTTTTTTTT
PBANKA_0829400	conserved Plasmodium protein, unknown function	G1	ACAAAAAAAAACA AATTATA	100	AGTATCGATTTTATTAGTATGTTCAGCTGTTTATTT TACTTTCAAAACATTAGCAAATAAAATAATAAAAT TCTCACATATAAGTGCTATAAAATTCATT	ACATGCGATTTTGATATAATATTTTAAAATTTG TGTGTACTATTATTAAAGCTATCTTATATTATTT TTTTCATTTTTTTTTATATTAGTATTTAATTTTT

Table S3: Knockout pools of 22X pPbHiT vectors

Target gene ID	Gene name	PlasmoGEM phenotype	Spike-in control in larger CRISPR pools	G1 sequence (5'-->3')	G2 sequence (5'-->3')	HR1	HR2
PBANKA_0515000	ookinete surface protein P25	Dispensable	Yes	ATATTACAAGAGCATATTGG	ACCGCAAACTAATGAACATG	ATATTTCCATTTTATACAATACATAAAAGCCCATAAAAAAAATATATACACTTTATTAATAAAAATTTTATTTTTGTTATTCGTTTAAATTTATTTAAAA	ATAAACAAATATACCTGGATAATTTTCACTAATTCAACCTTAACTTTTAAGTTTAAACGCTTTATAGTTATATTTTTGTGGGCAATAAAAAATATATATA
PBANKA_0933700	mitogen-activated protein kinase 2, MAPK2	Dispensable	No	ATTGATTCAAAAAAAAAACG	CAGATTATGTAGCAACACGT	TTATCATAAAATTGTGCATTAACAGTTAGAAGAGGATTGCCATTTTTTGTTTTTAATTTTTATGCTATTATTTTTTTCTTAATTTTTTGGACAAAAAAAA	TTTCAAATTATAATTACTCGAAAATAAATATGTATGTATCATAAACTGAATTTTGGGGTAACATATAAAAATAAATAAATGTAAAAAATAAAACCATATT
PBANKA_1013300	mitogen-activated protein kinase 1, MAPK1	Dispensable	Yes	ATTGATTCAAAAAAAAAACG	CAGATTATGTAGCAACACGT	TTATCATAAAATTGTGCATTAACAGTTAGAAGAGGATTGCCATTTTTTGTTTTTAATTTTTATGCTATTATTTTTTTCTTAATTTTTTGGACAAAAAAAA	TTTCAAATTATAATTACTCGAAAATAAATATGTATGTATCATAAACTGAATTTTGGGGTAACATATAAAAATAAATAAATGTAAAAAATAAAACCATATT
PBANKA_1037800	secreted ookinete adhesive protein, SOAP	Dispensable	Yes	TCGTGAAGATGCCTTAACAT	TAGTCCCTTTGCATGTGCGG	TAAATATTGCTTACATATTCCTCTCTATAAATATATATATTCTTTTTAGCAATATATTTCTCTTATATATATACCCTTTTTTGTATTTATATTAATCAAA	TTATGTGTAAATACTGTATTATTATAAGGAAATATTTATTTATCAAAAAATATCAAAAGAAAAAATTATTTTTTTGTGTGACTTAGTCCGCATTATATAT
PBANKA_1034400	plasmepsin IV, PM IV	Slow	Yes	TGACATAAAACTATTCTGAG	AAAGAGTCAAATTACTCCAA	AATTTATTTTTTTTATATAATCCGTCATTATTTTTACATTATTATGCTTTACAACATATATATATATCCAAATTTTTTTTTCTCCTTTAATTAGTTCAAA	ATAAAAATAAAAAAAATTATATATGATATATTACACGTACCATAACATGCCTGCTTTTATATATTTTATGTAAGTATCATCATATATATTTTAACAAAAG
PBANKA_1101400	rhoptry-associated protein 2/3, RAP2/3	Slow	No	TGCTTGAACCAAATCGTCGG	ATCATCTATAAACTTTGCAG	AGTTATTATTACTTTATTATTTTATATATATATTTTTATTTACAACGCATATATTAATTGCGCCAGTTATATTTTTATATAAAACGAAAGTGAAGGCAAA	GTTTTTTTATCAATAAATGAGCTATTAAGTTTTTATTTTTTATGTAATAGTTATATATATATATTATGTTTTTTTATTGATGATATGTTATTTAATCTAT
PBANKA_1104200	2-oxoisovalerate dehydrogenase subunit beta, mitochondrial, putative, BCKDHB	Slow	Yes	TGGGGTGCAGTAGGACATGG	AAAAATAAAATAATTCAAGG	AATATAATGTATCCATATCAATCATAGTGCCGCAGTTTCATTTAATTAGTGTTACTATTATTACAATTTTTATTTTTTATAAATCCGATTAACATATAAA	AATTACAAAACTAATTTTTTATTATCTCGTTTTTAATCATTATACATAACAAGGAAATATAATTTTATCTACCCTTATTTGTGTAAATCACAGCTTTTTT
PBANKA_1401600	methyltransferase, putative	Slow	Yes	ATTGTCGCTTCATATGCTGG	GTACTAGTCATAATTTCATG	CACTTCTTCATGATCACATTGCTTTCATAAGAAGCTCATACTTAATCAGAACCATATCAGGTGTAGATAATTATTTTATTTATTTAAATACGTGTTAACA	TAAATCTTCAAATCAAACACATGTTTACCTATAACATATGCACACATATACTTAAAACATGAAATATTATTAATGTATAACATAGTTTTTATTACATTTC

PBANKA_0211000	mitochondrial import inner membrane translocase subunit TIM50, putative,	Essential	No	CAAGAAGTAATATCAAAGTG	ACAACGCCAAAAAACAACAA	AGTATTTAAAAATACTACTAAATTTGATTTTTTTGCTATTTTTCTTAATGAAATTATAAAAACAACATATTGAAAAAGTGGTGAATGAAATAAAGATAGA	AGGGAAAAAATTTGGGAAAAAATTTGGGAAAAAATGAGAAAAAATTTGGGAAAAAATATGAAAAAATGAAAATATTTTAAGCAAAATGCATATATGTATA
PBANKA_0706400	conserved Plasmodium protein, unknown function	Essential	No	TTGGCGAAAGAAGAGGAAAG	AAAAAGAGAAGATTTCAATG	CATATGAAATAATTTTTTAATTATGATTATAGTCAAATTATAGTTAATTAGAAGTAATAATTATATATAAGGAACTTTGAGAATACGAAGAAAAAAAAAC	TTTAATGATATTATTTTATTTCATTTCATTTTTTCCGTTGTCTTTTCTTGTTTATGGCATCTCATGGTGTACTACATACCGTTTGGTTTGTCCACGTTGA
PBANKA_1039700	cytochrome c oxidase subunit ApiCOX19, putative	Essential	No	ATAGATGATTTAAATAGTGA	TGACCATAAGAACCAAACAA	CTAATATTGAAGAGTAGAAAGGGGAGAAGATTACAAAGCCAATAATTTTCAAAAATAATCACAAGTTCAATTATATTACCATATATGTGCGAACACAAGA	ACAAAAAAATTGTAGTAGCATATGCCTACAAATTCCTTTTAAAGCAAATGCCCTTATATGCGCATTCCATTCAATTGTTTATGTTTATACTCCACTTTGTA
PBANKA_1214100	tubulin binding cofactor c, putative	Essential	No	AAACAGAACTTAATATGTCA	ATATCATATTATATTGCATG	ATCATTTTTTTTATCAATATTAAAATTGGTGTATCCTTTTACAAATATACAATTATTAATATACTTATTATTTATTTGTTTTCAAAAAAAAAAACAAAACA	GAAAAATAAACATATATTTTAAATCACCCTGGTGTAATATGCATATGTGTATTATATAATCTTGTAAATATCAATTTTTTTTTTTTTAATTTTTCCGCAA

Table S4: Knockout pools of 48X, 96X and 192X pPbHiT vectors and spiking controls

			Target gene ID	Gene name	PlasmoGEM phenotype	Guide 1 sequence (5'-->3')	Guide 2 sequence (5'-->3')		
From pool of 22X knockout vectors			PBANKA_1013300	mitogen-activated protein kinase 1, MAPK1	Dispensable	ATTGATTCAAAAAAAAAACG	CAGATTATGTAGCAACACGT		
			PBANKA_1037800	secreted ookinete adhesive protein, SOAP	Dispensable	TCGTGAAGATGCCTTAACAT	TAGTCCCTTTGCATGTGCGG		
			PBANKA_0515000	ookinete surface protein P25	Dispensable	ATATTACAAGAGCATATTGG			
			PBANKA_1401600	methyltransferase, putative	Slow	ATTGTCGCTTCATATGCTGG	GTACTAGTCATAATTTCATG		
			PBANKA_1034400	plasmepsin IV, PM IV	Slow	TGACATAAAACTATTCTGAG	AAAGAGTCAAATTACTCCAA		
			PBANKA_1104200	2-oxoisovalerate dehydrogenase subunit beta, mitochondrial, putative, BCKDHB	Slow	TGGGGTGCAGTAGGACATGG	AAAAATAAAATAATTCAAGG		
**Larger CRISPR pools**									
**Pool sizes**			**Target gene ID**	**Gene name**	**PlasmoGEM phenotype**	**G1 sequence (5'-->3')**	**G2 sequence (5'-->3')**	**HR1**	**HR2**
192X	96X	48X	PBANKA_1459900	signal recognition particle receptor subunit beta, putative (SRPRB)	Essential	TTTCGTCTTACCACGGGTAT	TGAAAGGGCTCCAATACCCG	AATAATAAAGTTAGTACTAACAAAATTACTAAAGGTATAACTAGCCTTCTGTATCATAATACAAAATCCAAACATATAGATTTTTTTTAATAGACATAAA	TTTTTATTTCATTAAATTATTATATTTATTCATTCATATACTCGTTTTTTATTAATATATTTATTAATTTATTCTATGTTTATATTTTTTTTCACCAACA

			PBANKA_0804000	60S ribosomal protein L37, putative (RPL37)	Essential	GGTAAAGCTGGAAAAGGTAC	AGAAGAAATACAATTGGTAC	AAAAAAAATAATATTTATTTATGTTATTTAAAAAAAAAAAATATATAATTTTACTAATAAATATTAATTAATTTATATAATTTTATTATTTAAAATATAAA	TTGTTATGTAGATATACCCCATCAAAAAATTACCAAAATATACTATATTTGCTTGTGACTTAATTTTTTGGGAATAACGATTTAGGCAAAACGAGAACAC
			PBANKA_0809200	ribosomal protein L35, apicoplast, putative	Essential	CAACGTAGTTAAACATAGCC	CAAATAAATCGATTGCAAAA	AATATTGATATGTTCATATTTTATTTACACATTTTACAAGGTTATATATGGGAGATAATAGAAAGTGTCATGATTTGGATACAAAACTTTCGAAATAATT	CAGCACATGTTCCAATATATCTGTACACATTTTTATATATTAAACATATTTTTATTTTTTCTGTTCCTTTTTATATATTTATTTTATATGTTTCTGGAAT
			PBANKA_0703200	ribosomal protein L21, apicoplast, putative	Essential	AAAATTGAGTAAAAGGCCTG	ATAGGATCAAAATCATCCAG	TTTTAAAAAAGAAATGAAAAACATTTAATAGTATATTTTCGTATAGTTAAAAATGAGAGAAACAAAAATATGTAAAAGGCTAGAATGAAAATGTGGAAAT	TGAAATATATATTATGTGAATAATTTTTTTTTTTTTTTAGGTCTAGTTTTAGGCCTCCTTCTATGTTTTGTCTTTTTTTTCTGTGCAAAATTATAATTT
			PBANKA_0505400	conserved Plasmodium protein, unknown function	Essential	TAGATTGAATGGATCAGAAG	GTTGGTACATCTGTATTGTC	AAAAAATAAAAGAGAGAAAAGCACGACAAGCCAAACAGTAACAATAATAGTAGTAATAGCTAGCTAAAAAAAAAAAAAAAAAACATATCAGAATAATAGA	GAAGTGTAGCTAGCTGATTTAGGAAAATAAATGCAGTTATTACCACATTTTCCTTATTCAAATAATTTTTTTTATTTTTTAATCCCGTAACTAGGTAGTA
			PBANKA_0812300	spindle and kinetochore-associated protein 1, putative (SKA1)	Essential	CATTTGGCTTCCCAGATCTA	TAAGTCTTAATTGCTCATCA	CCGCAAAAGAATACCCCAAAACAAAAAACCTCCCTTTCAGAACAGAAATGTTTTAATTTTATTACATTTATGAATATTATTAATAAAAATATTAAAAAAT	GAATATCAAATTGTAATAAATACATAAAAAGATTAAAAATAAAAAGTATTAATACATACAATTTGAAGATAAAATAAAAAATAGTAACGTGCCAAATTGA
			PBANKA_1009800	cytochrome c oxidase assembly protein COX15, putative (COX15)	Essential	GGATGGTGGATGGTTAAAAG	AATTGGTGCACTAATGCCAG	GTGATGAATATATGCTTGTGCACCTCTCTCTCTTTAATACAGGTATATACCCTTTTTTAACACAATTATTTTTAGGAAAAATTGAATAAAATAAGGAAGT	ATAGTTGACACTCCTTTCTTTCAAGTATATATACTCAAACATTAACATCGAAGCGTTAAAAATATATGCGTTTGCCAATTTGTAAACATATATTTTTTTA
			PBANKA_1136500	conserved Plasmodium protein, unknown function	Essential	AAAATATGATAAAATACTGG	GTACCCATTGTAGATTTGAA	TTTTGTTTGCACCTTAACTTTTTTTATTGACAAACGTGCATGTTTTATAAGAAACCCTTTTTTTTACATCGATATATTGCTACAAAATACCAATAAGAAT	GTTAAAAAGGAAAGTGTGAATACATCATAAAAAATTTGCGCGGAGAATTATAATGTATGGGAAGAAAATAATGACGGAATTTTTTTACTACTTGCTAATTA
			PBANKA_0914900	onserved protein, unknown function	Essential	TTTACGGTGAAGCGATAATT	ATGAACGCGTCATAATAGTT	TTGAATGCTGTATATAAGTGTAAATAAATACAAGTTTTTCGAGTTAGAGAGTATATATCAAACATGATCTACT	TCACATATAGTTTGCAATACATTTTTGTGTATAATTTTTTCATGTTTACATTTTTGTGTGAAAAAGTAATGCGTGAAAATGTTCATACATATTTATATAG

									ATATATATCAGTCTTCAACCATAAAAA	
			PBANKA_0817800	conserved Plasmodium protein, unknown function	Essential	CGAAAGCGAAGAATCCCCAA	AAACAATTCCTGATAAAATG	CATATTATACTGAGGATTTATTACTACAAAATTAAACATTTATCATATTTTAATTGCTATAATTTGTTACAATTTTTCAATTTTTTCAACTTTTTTTAAA	AATAGAAGAAAATAGCAAGGAAATGATACCCCAGAGCGCGAAATATCCAACAATGGAGAAGCAACTATGGAATATGGAAGAACATCCATATAAAATTATA
			PBANKA_1233900	50S ribosomal protein L14, mitochondrial, putative	Essential	GTTAGGTGTGCAGATAACAG	ATGCTTTATGTGGCCTTTGG	ACTTTGAATTCCAAAATAAAAAGTTATAACACATGTAATATATGTATATATTGGATATACGCATGCTTGTTCATGTGTGTGTGAATTAAGATCTTGATTA	AGTATACTCAAAATTTTTTGTTTCGAAAAAAATTAATGTGCTACATCCTTACATATAAGAGTATTTAGGCGTGTGCATATATATTTACCACTATTTTGTA
			PBANKA_0514900	ookinete surface protein P28	Slow growers	TTTCTAAGCCAAAATTTCCC	AAACCCCAAGCACCAGGTAC	ATTTATATTCTCATAATTTACGTAAAAAAACAAAACAATTTCATAAAATTATATCATAACAGTTATTTTAACAATTATTTTATATTAAATTTTCACGAAA	TATATTCAATTGTTATCGCATATTGTAGGAATATTTATACATATTTATATATAGAGACACAAAAAAAAAATTGAAGCAAATTTGACTTTTAAATAATTTA
			PBANKA_1362100	tyrosine kinase-like protein, putative (TKL3)	Slow growers	TTTATAGGGGTAAAGGTATG	TAATGGATCTGCATTGTCAA	AAGTTGAATAGTATAAGTATTGAAATCCAAATGTATAAAAATATGTAAGCAATGTGTGCTTATATGGATTAAATATGAATAACTTAAGTTTATTAAAAAG	AGTTAAGAAATAAAACGCATGCTTAAAAAAAAAAAAAAAATAATAAAAAAATAAAATAATAATAAAAAAAACTTTCTTTATTCGAAAAAATTTGTCGAAAT
			PBANKA_1108400	single-stranded DNA-binding protein, putative (SSB)	Slow growers	CCTATAGACAAAGATGAAGG	CCTCCTTCATCTTTGTCTAT	ATAAAATAAATATAACGTGATATTGGATAATTTCCATATGCATTATACATATATATGGATGTGCCATAGTTATAATTTGTGATAATCGTACAATAAAATA	TTTTTGCAGAAAAAAAACGTATTACCATGCAAGGTGAATATTTTTTTTGTTAAATTTAGCTAGCTAACTAGCTATTTCATATGAAAAATGATATACACAT
			PBANKA_1322400	exonuclease V, mitochondrial, putative	Slow growers	GTTCGAAATTATGTACTTAG	AAAGTGCCAAATTCGAAAAA	TTTTTTTAAGATTATAAATGCACACGCACACATATATATATACATTTTTTTGTTCGAGATATATATACCTTGCCACCTCTACATCAATAGGAATATAAAA	ACAAAAGTTACAATTTGAATCATATTAAACTATAAAATCATAAAAATAATTTATCATTTGCACAAATGTATTATCTCTATGTGCGCATTTATATATGCAC
			PBANKA_1426400	mitochondrial carrier protein, putative	Slow growers	ACAGTTCCTTAACAAATGCG	GAATTACCACGCATTTGTTA	AATAACATAATATAGGCGTAAAAAATGAAGATTATAATTTTGTAACTTAATTGGAAAATATAAAAATATATATACTAGCCAAAAAGATGAAACGTGTAAA	GGACATGTTTTACTATGCATACATGCACTTATTGTTTCTCTATGTTTTCCGTTTATGATGCTTTGTTTTTTTTAAACTTATATAATATGTAAAAATTTTA
			PBANKA_0314200	calcium-dependent	Dispensable	TCAGTGAAGAGAGGCTAAGG	ATGGAATGATGTCTTAGGGG	GTGCATGTATAAATATATACACATACAGAAATATATAAACCATTGAGTCATCCCTAACTACCCAAGCATAATT	AAAAGAGGAATAGAAATAAGAGAGAAAAAGAAAAATAATATTCTAAGACATTTTTCTGGCTAACTAAAATAA

				protein kinase 1 (CDPK1)				AGACAGAGTGATTTAAATTAACATGAA	TATATTTGCTATACCATATAGCTTTTTA
			PBANKA_0616700	NIMA related kinase 4 (NEK4)	Dispensable	TCCCTTTGTTGAATGAAATG	ATTGTAATGAAGCATTGTAA	GATTGTGTATTGCTCATTCTTTATTATACATATTCTCACAAAATTTTTTTTATTAACTAATATAATTTCCTGTAACCTTACAATTTGAAATTCCTAAAG	TATGTTATTCTTCCACAAAAAAAAAGAATATAATATTACAGTCCATACACAGGCATATATATATATATATATATATATACAAATGATTGATAAAAAATAT
			PBANKA_0408200	calcium-dependent protein kinase 3 (CDPK3)	Dispensable	ACACCCCGTTAAAACTTGAG	GAAGTTGACAAGAACAATGA	ATTTTTTAAAATTTGTTCATTTACTTCTTTAAATTTGTCCACTTGCCTTTTTAAATATATACTTTTATTTTTTATTTTGGAAATAGTTTAATACGTAAAA	ATGGAACTATTGATTAAAGAATTATAATATACAGGAACATATGATTATTTATTGGTTTTTTGTAAACCTATAGTGTTGTAGACTCTTGTTTTAATGAGGC
			PBANKA_1305200	serine/threonine protein kinase, putative	Dispensable	TCTATGAGACTTCTCACTTG	GTATGAGTTGAATTTGATGG	ATAGTTACTACTTTGTTCTAATGTTTATTCCTTAATTCGCTTTTTTTCGCGTCTTTATTGTATCGTTCCTCCTTTTCCTTTTTGGAGTATTGATTTTAAA	TTAGGATTAAAACTAAGGAAAAATTATATTCATACCTTTCTACGTGTATGTTAATAATATACAAGGAAAGACCCTTATATATTAATGCTATAATTTTATA
			PBANKA_1414500	glycogen synthase kinase-3 alpha, putative (GSK3alpha)	Dispensable	AGGGCACCGGAATTGCTTTG	AAAGGAGAAACAAAGTTTCT	ATATAGACACAAATAATAAATATAAACTTATTTATTTGTGCTATATGCATATATCGAAGAAAAATATAGTGATTAAGCTCATAAAGTATATAAATACAAA	AAAAAAAAAATAGGATGCATTAAAATATATAAAAAATAAAATATAAAGAGATAGACATATTGTAAGAAAAAAATACATATATATTAATTTGTATAAGAAT
			PBANKA_0926600	protein GCN20, putative	Dispensable	ATATGTGGTGTTAATGGTAG	GCATTGAGTTTGTATAAAGG	TATGATAAATATACCAAAATAATATAATTATTGAAATAATATTCTCACAAAAATCGATATATATAAGGATTTATAATCACACATGGATATATGGGGAAAT	TTATATACAACTTTCATATATAAATTTATAGCCATTAATTCTATTTTTAATATATATGTTTGTGTAAATACATATGAAAACGTGGGCATAATTAAAAAAT
			PBANKA_1352600	serine/threonine protein kinase, putative	Dispensable	TATGTAAGCATTTCCAATTG	TCCGGAAGTCCAACGCCTGA	TTATTCCTTCACGAATGTTGGATTGTTTCATTTACTTGGCTAGTTATTATTCAACTATTTTTTTTGTAATATAATATAATTAAAAAAATTTTCAAAAAAA	GAAACTTTCTTGACAATTATATGACTTAACTAGCGAAATTGTATTTTTATTTTATTATTTTTTTTTTGTTGTATTTACCTGCACGTACATATTTTATTT
			PBANKA_1421600	calcium/calmodulin-dependent protein kinase, putative	Dispensable	GATCGAACCCTTGATAATAA	AATCTAACTTGAAATAATTC	TTTACTTGGTCATGTATTCTTTATTTTTTTTAATTTATTTTCCCGTTTGGATATAATATAGAATAAAGCATACACAAGCATGCCTTTGGAAAAAATTCCG	ATATTATAAGTATATTTGATACAAGCTTATTTATCACATATAAATTTAATATTTATTTATAATTAAATCAATATGACCATAATTATTTCCATTTAAAAAT
			PBANKA_0308500	tyrosine kinase-like protein, putative (TKL1)	Dispensable	ACTTGAGCTATGATGTCTAG	TATTCGAGTGCTATTGCTTG	GGCATATATATACATACATATGCGTAAGCATGGTAATTGATAGAAGCAATGGTTCAAAACTGTTTGAACAA	GCGTTTTCACTCCTTTCAGAAAGGACAGACATGTATGTTTACAAATTTTAAACTTTTTTCACATTTTTTGGAG

								GCACAAATGAATTATATAATTTAACAAAA	AACAATAATGTTTTCCTCATGTTTCAT
			PBANKA_0604400	targeted glyoxalase II, putative (tGLO2)	Dispensable	TGGCTTGCCTATATGCGCAT	TACATTGCCTATGCGCATAT	GAAGGATGCCACATTTTATTTAATATATTGTTTGATTATTTTGAATGTGTTTGCACACATATACTACTAATATAACAATTTCAAGGTATGATATTATAAA	CTGTTTTAACCAATTAAATGAATGTACCAAGCATATGCGAAGTGTAAATATCATAATTGCTTGCTTGTTTCACACTTTTGTATACATTCGCAATATAGAA
			PBANKA_0103700	conserved Plasmodium protein, unknown function	Unstudied	GGCAGGAATAATTCCATCAG	AAAAGAAGAAAAGATGACAA	ATCATATCCCCTATAATACATATAATTTAATGTTTACCATTTAAAAAAATGCCACTATATTGATACTTTTAAAAGACGAATTTCATTGTAGAATAAAATG	GTTCCAAATTTATAATAACCATTTTGTTTTTTCCGTATATATGTGAGTAATACTATTATGCATACTTTTGTAAAGACATAACAATTCAATAAATAATTAT
			PBANKA_0111400	conserved Plasmodium protein, unknown function	Unstudied	TTTGTTGTCGCGTTTCTTTC	GCTATTTGACGTCCCGATCT	GTATTAAATTATTATATAGATATATAAATATATTTTTTTACTTAAAAGTTTGAATTACTGAAAACAAAATTATACTATTTGAAAATATATAGGAGGGAAA	AATGCCTAAGAGAAAAGAATTTATGTAATTCGCTTTATATATTTTTTTTAAAGTATATTTATTCATTATTGTCAGAAATAAATCTAAAATTATTTCATAG
			PBANKA_0111500	conserved Plasmodium protein, unknown function	Unstudied	GCTCATTGTTGATAAGACTA	GTTCCTGTACTTGTTTAAAA	TCTTTAACAAATAAATAAAAAAATAATATAATTTTATATTACATTACATAAAATAACAAAATGATACAACATTTAGATTATAATATTTAACAAAAATAAT	TTTTTGTATTTTTTTTATTCATAAATAGTAATATGATATCAATTTTGTAAACATTTATAAATAAACATTTTTATTGTAGTAAACCGGGTATATTATGTTT
			PBANKA_0210800	conserved Plasmodium protein, unknown function	Unstudied	TAATAAAACGCCATGTATTT	TCATATAACTCCTAAATACA	TTAAGGCTACAATATTGTTACAAAAAAATAAAAAAAAGATAAAACAATGTAATATTTACCACTTAGACCACAATTTAAAATAACGATAAATATGCAATAA	AATTGTATCAAACGTGAAAAAATTAATGAACATCTTTATGCATATATTTTTCATCATTCTACATAGTTGGGGCTGTTTATTTTTTTTTCATTTTTTCAAT
			PBANKA_0315700	conserved Plasmodium protein, unknown function	Unstudied	ATATACTCGAAGGATATTCT	GGATATTCTTGGCGATTTAA	ATTTTAAAAAAACATAAATGCGAGTATCATTTTCTTATTATGTTTACGCAAACTTAATTTTAATTTTGTACTAATATTTCATTATTCTTATTAATAAACT	CATATTTATTTTACAAAATTAGCATATTTATAGGACTCCCATAACACCAATTAAAGACAAAATATTAATTAAAATTAATCGACATATTTTTTTTAAATTA
			PBANKA_0408800	conserved Plasmodium protein, unknown function	Unstudied	CTGGGTAGTTGCACATATTT	ATTTGATGAGTAATAGGTGC	ATTCATTGATATTTCTCGAAACATCATACACACCTATATATATTAATCATATCTAATTGTATTTCGAAAATTTTGCATTATTACATAACATATATATAAA	AAAAAAACTGAAGAAATATTATAGTAAAACATTTAAATAGTGGAATAAAAACAAAAAAATATAATTTGGAAATAATCCTTTATAAATTATGTATGTGGAC
			PBANKA_0409000	conserved Plasmodium protein,	Unstudied	CAAACTGCCAGATCAACTTC	TAATGCGATCCAAAATTCCA	CTATATATATATTGGGAACATAAATAGATAAAAATTAAACAATAACTAAAAAGACAAAATTGGAATTAATAA	CATATTTTTAAAGCAAAAATTATTTATATATTTTATCTTTTTTTATATATTT

				unknown function				AAAAAAATACATTCTTTATTAAAGTATA	TTTCATTCTTCATATATTTTTTCTATCATTTTTTTCTTCATTGTCTAT
			PBANKA_0502900	conserved Plasmodium protein, unknown function	Unstudied	ATTGTCTAGAAATATGTGTG	CATGTTCTCAAACTAATGA	CACATTAATAATAGTGAATATGGAATTATTTTCCTAAATGTTCAACTACGTGCTTAGTATTTATTTATTTTATTTATTTGTTAACATTTGCTCGCAATAT	TTTTTCAAAATACTACATTAATATTGAATTGGCTCAAATTTGTATAACACACAAATAATATATATATATATATATATATATATATTGTATACCATTTAAT
			PBANKA_0509600	conserved Plasmodium protein, unknown function	Unstudied	TATGGAAATATGCTTATTTC	GTTTGCATCTGAATCGAGGC	GAATAAACACATAGGCATATATATATGTATGCATATTTTGTGTTTTTTATAATTAAATTTAATTTCGAGCATTTTAGTGTATTTTTTTTTGGAGATTAAA	CAAATCTGTGCATATCATTATACGGCTATGATTGTGGGAAAAAAAAATTTAATCGAACAATACACAATTAGTTAGTATATGCAATATATTTATTATTTCA
			PBANKA_0519100	conserved Plasmodium protein, unknown function	Unstudied	TCAGTTTCGGGACTTACACC	GGTGGTGCTGGTGCTGTTCC	TGTTCGCATTTAGAAAACAGAATATTATTATAAATATATTATATCCTCTTGTTTAATTTATTGTAAATTTTTGTGACGTATTACTATAAATTATATAAAA	ATGTTCTAAAAAATGTCAAAAAAATGAAATATTTATCATTTTTTAACCTATTTTTTTAATATATTACCATTAATATATTGTTATTAATTTATTGACTTTT
			PBANKA_0519200	conserved Plasmodium protein, unknown function	Unstudied	ATTCTAGGCAAGATGCATCA	AATCTAGACAAGATTCATCA	AATTATAATTTTTGCACACAAATAGTTATTTTACCTTTACTAGTATTACAACCACTATTGTTATTTTATTTTATTTTATTTTTTTAAACATAAATTCACA	GAGGGGATATGCATATACATATATATAACAACAGGCGATAAATGAAGAATTTTAAGGTTATTTCATTTATTTTAGTAATTAAATATAGCATCTCAATTGT
			PBANKA_0519400	conserved Plasmodium protein, unknown function	Unstudied	TCAGTTGAAAACCCTTCAGG	TCTGATCCAGGGACGCCTAA	AATAAATGTGTAATGTACTATGCATAGCCATTTTTGCGAAATAATATATATACATACATGCATACAACTTTTTGTTATTTATTGTTAAATATATAAAAAA	TAAGGGGAAAAGTGCAAACTCAGAAATTTAAAAGTAAGAAGGCTTGTTAGTATGTACCTATATATATTTATATTGGTCACGTTACGAAGTGATCATTAAA
			PBANKA_0523700	conserved Plasmodium protein, unknown function	Unstudied	TATCATCCACAGACGAAATA	GAAAGATATTCTAGAGAATG	AAATAAAATGATTAAAAAATATCAATTTTTATATAATATTAAAAAAATAACGAATGACAAATGAATATATATAAAGTATATGCAAATAATCAATTTAAAA	AAAATATTTCATAAATCCCCTTTAAAATATCACCTACCACATATATAGAATAAATTATTTGGAAAAAAAATAAAATATTTAATAGACATACATAATACGC
			PBANKA_0524100	conserved Plasmodium protein, unknown function	Unstudied	TGATCGGGCCCTATTACTTC	TCGTTATATCACAACTCAAG	CTACCACAAAAATATAAACTAACAGTTAAACAATATATGACAATATGTCATTTTGTATACAAATGAATTATAATAATTAGGTATATTTTATATTTGTATA	TATAATAGTAAGTAAATATTTATTTGACCAAATTCGTCTGTATTTTTTTATATATCCATAATTCTTCATTAAAATTAAAGGGATTTTCAAAAAAAAAATG
			PBANKA_0603650	conserved Plasmodium protein,	Unstudied	AATGGTGTAAATATTAACCG	AGAGGAGCTAACACGCCGAA	ACTAACAAATAATAAATTATAAAGTATTGTAACATAAGTGATGGGGAAATGTGTATAAGTTGCCAAAAATT	AATATAATAAAATAAAAATAGGAAATATATAAAATTGTATATTATGCTAC

				unknown function				TATTTTATTTTAATTTTTTTGAGTATAAA	TACATTAGTGAATTATACATTTTTAATAAATTTATAGTCTATTGTGTATT
			PBANKA_0619000	conserved Plasmodium protein, unknown function	Unstudied	GTTTGTTTCGATCCTTCAGG	AATCTAGAAAATGAGAATTC	AAATTTTCAACCAGGTGTAATTATGGCATTTAATGATTAAAATATTTGATAAGAAAAAAAAAAATAAATAAAATCCTCAATTTTCCAAATAAAATAAAATG	ATTTAATGCAAATAAAATGTGTGGCATAATATCGCAAGTACCAATATGCCTTTGGCAATTTTATATAAGCACCATTTTCGTCTTTTTTATTATCCTCAAA
			PBANKA_0705800	conserved Plasmodium protein, unknown function	Unstudied	GTTGAGGAGCAGCTTGGTAT	TTTAGGGGTAGATATAGAAG	TATTATTAAAAATAATAATTTAAAAAATTTGTAGTAAGCCTATTGTTTTCCCAAACTATGTACGATTAAGAAAGCTTGCTAACCAACAATAAATAATAAA	ATGTTGATAATACAACATAATATGGAAAATGGGAAAAATATCAAATATAAAAAAATAAGAAATGTAGGAAAGGAAAATACCGAAAAAAGGCTCAACATAG
			PBANKA_0709800	conserved Plasmodium protein, unknown function	Unstudied	AAAGTGCGAGTATTAATTAT	GGACAGAAAGTTGAGTAAAG	TATATAGACAATATATAAAATTATTAAATAGCTGTTCCACTTGTACTTGTTACATATTTATAATTATATTTACAGTTGTACAATCATTGTGAGTGTTAAA	CAATAACAATTGTATGGAAGGAAGTATAAAAGATATCATTATTTTTCATATTTTAAAATATCGATATCGTTAAATTGTTAAAAAATGAAATAATAATAGC
			PBANKA_0715300	conserved Plasmodium protein, unknown function	Unstudied	ATTGGATCGATAATTTCTCC	TCCGGGATTGGCATTACATT	TATTATTTATTTTCTGTTTTATTAAACGGCTGCATTCCTTGACATACAGTATTGAGATCAAACACTACCTCTCCTTCCATATTGTACAAACTAATATATA	AAAAAAATATATAGCCTTAATAATAATAAAAATTAAAAAAATACTAAATACATAAGTATGAACAATTCGTATTTTATCTGTTTGATGGTTTGGCCCATTC
			PBANKA_0812900	conserved Plasmodium protein, unknown function	Unstudied	ACAGGCATACCACTTATAGT	CCATATGAGCCAACTATAAG	CGTGATATTATAAGGGCGTTTTAAAAAGCAGCATAGTAACATAGCTTTATTATTTTGTTGAATATTATTAAATGTACTATGGGGTGAATTATTCAATTAA	AAAAAAACAATGATATAATACACACATTTCCTCACGTTTTCTTTTTGTTCTTTTCTCGATAAACCAAGAGTTAGCAATAGTAATATATGCTATATTTGTA
			PBANKA_0817400	conserved Plasmodium protein, unknown function	Unstudied	TTCCGCGAAAAGTATTATAA	AATATTTAAATAATGACTAG	GGCCGAAAAGAAACGAAAGAAAAGGGGAAAGAGAAAAAAAAAATTTTTTTCTCTCCTCAATTTATAAAAAAAAGAAAAAAAGCGAGCTTGGCTTAATAAA	AGTTTTATCAATTAATCCGATATGCCCTATATAAAATCCAAGTGTATATGCTTTTTTTTATTTCTTTCCTTTTTTTTCTGGGATTCCTTACAGCATTTAT
			PBANKA_0821200	conserved Plasmodium protein, unknown function	Unstudied	CCTATTTCATGCTCCATCTA	CCATAGATGGAGCATGAAAT	ATCACTGTTGTTTTTATTTGTTCTTTGACAAGTATTCAAATTTGAATTTATATTCATTATTAATGATATACTAAGAATTAAAAAAATATAAACCATCAAT	TATTTGATAAAATTGTTTTATGTTTCCTTTTTTTGTCTGTCATTTTTTATTTTAAATAAATAATTAAAGAGAAAAAAATAATAATTTTGTGATATTTGCT
			PBANKA_0915400	conserved Plasmodium protein,	Unstudied	ATTATGTTATTAATACTTAA	TAACATAATTAAAGCAAATA	AAAGTACTAAGAGGTTACTTCAAAATATTTTAATTTAAAAGGATTATTAAAATATAGTCCAAAGATTATTATT	GGCCATCATTTTTTAATTGGGAAAGAACAAAAAATATAATAATGTAGA

				unknown function				CACAGTTATAATATATTTTTGACCAAA	ATTAACTATATTATTGTTATATACATCTGTATATTTGCAATTATTATTTTCT
			PBANKA_0930600	conserved Plasmodium protein, unknown function	Unstudied	GGTGCGGGAATAGTTATTGG	TCTCGAAGGTTAGTTGCTGT	ACAAACACTATATATTTATACATTATATAAACAAAAGTTACAAAACATGATATATATAATAATTTTTTTTAAAATATAGATAATCAAAATAGTTAAAAAT	CACATCATCATTAAATTTGTCAGTTTTAAATATCAAAAAGTAAAACAAACAAAGCAAAGAAAACTGTTTAATTTTGAAAAACCAATGCAGCTTTCAGGGT
			PBANKA_1015100	conserved Plasmodium protein, unknown function	Unstudied	TATTTGCTTGGATATTATCG	TATGAATTTCGAAAGTTTGG	AATATCCACATTGTTTCAAAATACAACATATTTATAGGCATATATTATTAATAAGCCCAAAGGGAGATATAATAACAATTCTATAAATTTTTTAACGACG	GTTATTTTTTTTTATTTTATTTTATTTTTTTTCTTTGTATTTTTTTTGTTTTTATTAAATAATTTAACTTTATTTTTATGTACAAAACGTATATTATATT
			PBANKA_1016900	conserved Plasmodium protein, unknown function	Unstudied	CAAAACCAAGTACACATTTG	TTGGCTAAAAGCTTGTGAAA	CTCACTATTTCCACTACAACAATACACAAATATAACCATATATATAGCAATATATTTTTTTTACATTTTTATTGTTATTAAACCTTGGAAGTAAAGTGCA	AAATGAACTTTGATGCATATAAAATTCAGGAATTTATTATTTTTTTAAGCTATTATTAAAAGCGTTTCAACCTTAAGTTAAAAAGGAGAAAAACAGGAAA
			PBANKA_1017200	conserved Plasmodium protein, unknown function	Unstudied	AATGTGCTACAATATTTAGC	TATGTAGCATCGATATCGTA	AACAAATAATAATTAAAGCATTTTTTAGTCTTCTTTTTCCTCGTTTATAATTACCCATTGTTTTAAATATCACAAATTTTGAAGTTTTTTTTTCTACATA	GTTCAGTTATAAAGCCCAATATTTGCAATTTTACTCCTTTGTGGATATTTAAAGTTCTTTTTAATTTTTTTTAAACAATATATCCCAGCATGCAATATAT
			PBANKA_1035000	conserved Plasmodium protein, unknown function	Unstudied	TTGGTGTTTAAGGATTTCGT	CGAATGCAACTTGGTGTTTA	TAAAAATAAAATATACATAGTTTTATAATAACCATTTAATTTGATCAGTCTTTTTTTAAAAAAATAAAAAAATAAAAATAAACCCCATTGATGTGTGAATA	ACAGTCAGGATAAGTTGTAAAATTGAAAACCCCATGAATAACCAACGGAAAACATTGTTATGAAATATACACACATTTGAAAAACCTTATCCAAGGCTTT
			PBANKA_1037300	conserved Plasmodium protein, unknown function	Unstudied	AATATATGTTCGTTAATTCC	CTCTCAAGAAGCTATTCATA	TTATATAGTTTCATTAAATTTTGTTTATTGTTATTTAACATATATTATACAAAAAGAAATATTTTTAAAAAATATACTCTAAAAATTTACAATCACTAAA	ACATGAAAGAAAACACCCATTTTTATGCTATTAATATACAATATAGACTAAATCATGAGTATACATAAATATATATACACCGATTTTATACTGACAAAGT
			PBANKA_1120000	conserved Plasmodium protein, unknown function	Unstudied	CAAACACGGAGAAATAGATG	GCAAACTGTTAAAACCGAAG	GTAATGATGTATACATTTGCATAAATTAATAAGCAAACAAATCGAGGGAATAGAATGTGAAAATATTAATAACTTAAACAATGAAAAAGACATATGGGGA	AACCTACTAGCCCCAATTTTTTTTTTTTTTCTGTCAATGACGCTTGTTTTTACACTTCTTATGTATTATTTATTTTTATAAGTTTATTAGTTCATTTTGT
			PBANKA_1134700	conserved Plasmodium protein,	Unstudied	CTGCCCATGGATTAGCTCAA	TATCACTGCTACAACACATG	CTGAAGTAATGCTATTGATTTTATATGATACAATTATTCCCAAATGTGCAGTTTAATTTCAGTATTTATGAAA	TAATATTAAGTGGAGGATGAAAAAAAATATTATTACAATAAATGGAAAA

				unknown function				AGTAATGTTATTTAATATTTTTTATAG	AAACTTGCATAATATAAGCACATACAAATATATTATATATGTTACAAAAAA
			PBANKA_1135300	conserved Plasmodium protein, unknown function	Unstudied	TTTCGACGATAGTATCTTCT	TCTTGGTCGCTACCTGAGTC	TTTAATAATCAATATACAAACCACTAAATTATTCACACACATCTATATGCTTACTTATTATAAAATATTTAGAGAGATATAGATACGGATAAAATACATA	ATAAAATAAAACAAATTAATGTGAAAATAGTTGTAAGAACAAAACTGCTTATTTAGTTATATATTCATATATTGTATATTTGTTTTAAATGACAAATAAT
			PBANKA_1201300	conserved Plasmodium protein, unknown function	Unstudied	AAAATCGAGGGTGTTAAAAG	GGGTGTTAAAAGTGGCGATC	AAAAAATAATATAAATATATAATGAAAAATAAAAATAATTCTTTATTTTAGCATATAAAAATATAAAATAAAATTAACTTATTGATATCTGTTAAAAAAA	GAACATCAAAAACAATTTCCACAATCTAATTATATATATTCTATTTAGTTACTATTTATTATATTTATTTATTTATTTATTTATTTATTTATTTATTTAT
			PBANKA_1206000	conserved Plasmodium protein, unknown function	Unstudied	TATATAGAAGGATTGACTGG	TCGGGCCAATATTATATTAG	TTAGCAATTTCGCAGTAAATACATGTACACACACACATTCCTATAATTCAACGTTTTATAAAAAACAAATAGAAGTATATACAAAGGGAAAAACAAAATA	TTAATTTGATAAACATCATTCTATTTATTTCCCAATCATTTATAAGTTATGCTTTATTTTATTAAATTATTATTATATTTGTCTTTATACTTTTAGTGTT
			PBANKA_1211700	conserved Plasmodium protein, unknown function	Unstudied	TAATTAGTCTTCGTATCTTG	AATGGAACATACGAAACGAA	GTTAAATATATTGTGTGTATACATATGCGATTGTGTGAACTAGTGGGGTTGTAGTAAAATATAAATATATTAAAGGAATAAAAAAAGTTAAAAAGAAAAA	AATTGCTAATAATAAATAAACGGGCATCATGATGTTTATGTACTTATAAATCGTTTTTCACTTCGTCATTTTTTTTTGGAATTTTTAAACCTATATAAAT
			PBANKA_1237100	conserved Plasmodium protein, unknown function	Unstudied	ATAGTGGCAGCTAATCCTAG	TGTTTGTAGTGGTTTATTCG	TTCCGTGTAAAATTTGTGTAGCAAAAAAATACATATATGCAACTATACCATGTGTTATTTATGGAAGACAATATAATTGGGATAATTTTTAAAAATAAAA	GTATATATAATAGTAGAAGAAGAATGGACAAAATATTTTGCATATATGTAATTGCATATTTAGAGTTTATTTAAAATCGAATGAACTTAGGATATTCATC
			PBANKA_1321900	conserved Plasmodium protein, unknown function	Unstudied	AAATGATACTATACCTACAG	GAGCAGGACATAAAACCCAA	AAACAAATAATTTTTTATAATTTAATTAACAAACACATACATATATTGTGTTTAAAAAAAAATATATAAATTACAAAGCCACAATCTTTATCCTTAATGAA	AATAAATTATAAAAAAAAATTAAAACAAAATTCATTAAAAGACGTATATTATTCATTGGAGTTTACATATCTTTTATATTTTATGCTTAATGTGTTTTTCA
			PBANKA_1327080	conserved Plasmodium protein, unknown function	Unstudied	CAATGCATGCTAAATGTGTT	TATGAAATTAAATGAAATTG	ATAAAAAAAGCGTGAATTTATAGGGAATATTATTATATTATATATGCACACCTATCCAAATAGTTTATAATTATTCATAAATCTTTATAACTCATATAAAA	ACAGAATACATTCGATATTTTATACAATTACAATTATTATGTGAATATATCTGCAATATAGTAATGTCAAAATTAGGAAATTATTATAGTATAAATATTG
			PBANKA_1329900	conserved Plasmodium protein,	Unstudied	ATGAAATGGTGCCTTCACAA	TATTGAAGGAACAATAATTC	TTATTTTATGATTGTCATTGTATTCATTATACTAGCTTAATATTTTTGTAACATAAAAAAATAAATACTTAAATC	ATTTTATAAAAAAACAAAATGAAAGAAATGTAAAATAATTTAAATTTAT

				unknown function				ATTAATTAATATTAAATACACACATA	AATTTTTTATTTACACATAGGTTTTCTAATTATATGTTTTTTTTTTTTTTA
			PBANKA_1345100	conserved Plasmodium protein, unknown function	Unstudied	GAAAGCCAAAGAAATGATGA	TTTGTAACCTATAAAAGGTG	AAATATGTATATATGACTTTTTTGCTTGCCTTTTAAACAATTAATGCGAAGCATAAAAAATGGGATAATTGTATTTTTTTTTCTGAACTGTTCATAAAATA	ATAAAATTTAAAAATAAAGAAATATATTTAGAGTGTAAAACTAACTATATGGTCGACATACATGTGTGCAAAGATATGTACATATATTTATACAACATTT
			PBANKA_1354800	conserved Plasmodium protein, unknown function	Unstudied	ATCTTATTAAACATCACACG	AACAGAATTAGGCTATAATA	TAATATCTGAATGAGTAATAAGACAATATCAATAAACTATATATATTTTTTGCAAAAATATATGGATGTATATAATATGCGTTTGTTGGGATGAATAATA	ATTTATGGCATATTTATGATAATCTAAAAACAAAGTAAAAAAAAAATGGAATAATAAAAATTTTAATATATTTAAAAACTTTTTATTCTTTCATTCAAAT
			PBANKA_1360200	conserved Plasmodium protein, unknown function	Unstudied	AGTGAAGAAGCAAAGTATAC	GACGATGTGCTAAGTAGCAA	TAAAGGCTTTATTCATAAAAATAAGCATACACATATTATAAATATATTTTTGTTTTTAACTATTTTTTTGGTATATTTATAATTCCAGTATTTTTGAAAA	ATATCTCAATGCATATTTTTTTGTTTTGTTTTTATATTTATGTTATGTAATATTTTAGTAAAACACTCGACACATTATTGATAATTATGTTAAAGGGTAT
			PBANKA_1408700	conserved Plasmodium protein, unknown function	Unstudied	CCTTTCGCTTGGTATGCATC	AGTTTAGTATGCCTTTCGCT	ATTATATATAACAACAGCAGTCACGCTGAAAATAACGTAAAATCGAAATTTATGAAAATGACCTAACAGATATATAAAATGAATAGAAATTCACGTCGAA	GCGTTTTTTTATTCCAGTAACTATAGTAAAAAAGCTGTAAATACTTATATGCCCTATTATAATATATTTTTTATAAAATTATAATGATTAAAAAATAAAT
			PBANKA_1436400	conserved Plasmodium protein, unknown function	Unstudied	TAAACCCTCAAGGTCACCTG	ACAATACAAGTAAACCCTCA	AACAAGTCACAATATAATTATAAACAAAAAAAACGCGTAAAAATACATTGTATACAATTTTTTAATAATATATATTCTTCCATTCCCAAATTAATAAAAA	GGCAGTAATATTGAACTGCAACGAAACATATCCTTATTTATTATTTTTTTTACGCTATATATTGCATTTATTTTATTTGATTATTATTTTTTTCGTTTTT
			PBANKA_1437100	conserved Plasmodium protein, unknown function	Unstudied	CATCTGATTCATATGGGTTC	TGCAGGTGTATCAACATATG	AAAAAAATGAGGTGATATTAGAATTATATGCATATATTATAAAGTTATATATATAATTTTTGCAATATATAGATATGTGTATAGATTGAGAAAATAGCAA	AATTTATCTGAAAGCGATGGCTGAAGTTTGTACCTAAAAAACATTTCGACAAAAAGTGGAAAAATAAAATGACTAGTGATGAAAAAAATTGAGCAAATTA
			PBANKA_1437900	conserved Plasmodium protein, unknown function	Unstudied	CCAAAATCGTCAAACTCCGT	ATCGACACAACCAATGTTCA	ATTTTTGTACAAAGTACAAGCTAATTTTTTTTTTAATATTATGCATATATATAATTATAAGGGGGAAAGGCAAAGTTTAAATAAAGCTCAATCAAGTTAAA	TTATTTTCCTATTCGGAATTTTTTTTATTCATTTTTTTGCGGTATTTATATTCAAGACTTGCGCTATAATTCATTTAAAAATTTGCACACACAAAAAAAA
			PBANKA_1452500	conserved Plasmodium protein,	Unstudied	GCTACTTCTGATTCTTCTGC	AGAAGTAGAAGCTGAAGTAC	TTTTTTTTAAATATAATATTTTAGAAAAAAATCAAATAAATATATATGTTTATTAGTAGAAATATCATATTTGTA	AAAGTAAAAGAATAATTTTATATTAACAATGCATGTTCTTTAATTTTTTT

				unknown function				ATTTAAAAAAAAAAAAAAAAAAAAAAA	TAAAGTGATCGTTGTATATGTATTCTCTATATGTGATGCATCTTATTTTA
			PBANKA_1464000	conserved Plasmodium protein, unknown function	Unstudied	CTTGGAATAATTTGTGCTTG	ACTAGTCATAATAATTTCAA	ATATTAAAATTGAATTTATGCATAATTTATATTAGATGGGTAATTGTGTATCATTGCATTTTATACTTAAACTTTGAATGAATTAGTATTAAAATAATAA	TTATATTATTTGTAATTTTTTATCTGTTTTTTATATATTTTTTTGGGGGTATGTTTCATTTGTTAATTTTTTATAAAATATTTTGAAAGTTAAAAATAAG
			PBANKA_1234100	protoheme IX farnesyltransferase, putative (COX10)	Unstudied	TTTGGATATTTCATGCTAGG	GCTGCACATCCCATTAGCAT	TTACGAATTTATATGAACATGTGTTTATATGGATATATTATATATACAAAAAAAGGAGGATATATTAACGAGATAAGAATTATATAAAAATGAGAAAAGA	TATGCGAATACAAACAGAAAAATGAACTCATAAAACCATATTAATTAAAACTTTATATTTTAGCGTAGTATGTGTTTTGCTTATAATTATCACGGTTTAT
			PBANKA_0605000	thiosulfate sulfurtransferase, putative (TUM1)	Unstudied	TGTTTGATTCGCTCTTTATC	TATATTCGGAAAAACTTCCA	CTTTCAATATATGAATATATGCATATATGTGATATATTTTCTGCGAAAACAAAACTAATTTATAATTACTAAAACAATATATATATACTAATAATGCAGT	AAATATATATTCCTTTAACTTATCGTATGAGCAAGACAATGTACTTGCATCTTTAACTATATTTTTTCAAAATACTTTATTTGTTATTGTTATTTTTTAT
			PBANKA_0514600	tRNA N6-adenosine threonylcarbamoyltransferase, putative (KAE1)	Unstudied	GAAGGAAGCGCTAATAAGTT	GGTGCTATGATTGCTTATAC	ATTGAGCTAGAAGTATACGTTTCAAAATAAAAGCGGAATAATAGATATATAAGGTCCTATACACAAGCCTAAACACATACACATATGCTAGTTTCCAAAA	TGTCTTTAAAAAAAAGATACGGCATTATTTTAGTTACTATTCCCATAAGGATAACCATTTAATGAAATCACAATTTGTGATATATAATTTTTTGCTATTT
			PBANKA_1140700	ferrochelatase (FC)	Unstudied	CAGTGCATTCTGCTGATTGG	TATATATAAAGGCTCATTCG	TTGTTTTGTCATTTGCTTGTGTTTTCTAAAAATCGTATATATTACACATATTTGTTGAATTGTATCGAATTTATAGTTTGATATACTTTTATATTTAATA	AATTATTATAAAATTCTTTAACAAGAATAAATCAAAACATCAAAAAAAATTAAATATTCTCAAATTAATATTAACCATAACGAATATTTTTATATTTTTT
			PBANKA_0306400	2C-methyl-D-erythritol 2,C4-cyclodiphosphate synthase, putative (IspF)	Unstudied	GGTGCAGGTTCATATGATTT	AATGGAATGCGAATAGGGCA	TATTCTTTTATTTTACATATTTTCGTGAAACGTGATATATATATATATATATATATATTTTCTATAGATTTTTCCAAATAATTAGTTATCTTTTTAAAGA	CGTTTTTGTGTTTAAAAATTTAATGCGACCAAATAAAAATATATAAAATAATAAATGAAACAAAACCTAAGCAGTTTATTTCATCCAAATAATGTGAATT
			PBANKA_1111400	inositol-3-phosphate synthase, putative (INO1)	Unstudied	AGATGCTGATTATTGTAAAG	TAACTGATATTATAGTATTA	AACACACAAATTAGGCTACGCGATATAAATCGTTGCAAATATAAAACAACTATTCGCATAATAATTCTGTATCTGTTTATTTTCGTCAATATATTTATAA	ATGTAATGTGTTTTATATGAATATAACCTTGTATACATATAGAATACTAGTAGAAACATCCATTGATTCTTAAGTTAATAGTTTACATTATTTTATTTCT
			PBANKA_0828100	fumarate hydratase, putative (FH)	Unstudied	GCTGGGCCTGCATAATATAT	GGAATAGGTGCACAATTTGG	AGTTTGTGCTTTGTGAATAAAAAATATTTTTCTAGCATTAACATCACAGGGATTTTGTGTGAAATTTATTTA	TTTTTCCAAAAATATGTACATTGTTGGAAAAAAAAAACTCATAAGTTTTAAACATTTTCAAACAAATAAAATTG

								ATAAATTTGTAAATTTTTGAACTCAACA	GGCCAAGATTTTTTATTCGTTATCTTT
			PBANKA_1344400	AMP deaminase (AMPD)	Unstudied	CATCAGAATCGGGATCCCAA	AAACATCAAAGGATCATCAG	CGATATGAGCCAACAAATCGATAAACGTCAAATTGATAATAAATATATATGTTTATATGTCTTTAAAAAAAATTTATATACTTATAAATATATAAAAATA	ATGTTCACATTTTTATAATTCAGTGACAATTTGAATTTTTATAAAATGGGGTGCAAGTTATTTCAACTACTAGAGCAGTACATAAATAACACGAATTTAT
			PBANKA_0719300	bifunctional dihydrofolate reductase-thymidylate synthase, putative (DHFR-TS)	Unstudied	TAAGGGAATTGGAAATGCGG	TATGGGAAGCTAATGGAACA	TGACTATTTGTTTCAACATTTTTTGAGTTTTCGTTTTATAATAGTATTTTCATTTGTATATTGCTTATATATATATAAATACACACCTAAATGTTACAAA	TGTTCGTTTTTCTTATTTATATATTTATACCAATTGATTGTATTTATAACTGTAAAAATGTGTATGTTGTGTGCATATTTTTTTTTGTGCATGCACATGC
			PBANKA_1127700	nicotinate phosphoribosyltransferase, putative (NAPRT)	Unstudied	ATTGGTATACGAATCGATTC	GACCTAATGATGGCTGTGAT	CCCAACATTGACATTTCTTAATTTTCCGATATAATAAAAAAAACTAAATTCCGTTATAATATATATATATATATATATATATATATACAATAAGAACAAA	ACAATATTCGCTTATAAAAATGGCGAAACTTCAAAGATTCTTTCCATTTTACTAGTATAATATCACTATATGCATGGACTTTGTTGAATGAATAATTTTT
			PBANKA_1435100	ribulose-phosphate 3-epimerase, putative	Unstudied	GGAGGGCCAAATGACAAATT	GACAGTAGAACCTGGATTTG	TTCATCAAATCAAAGGAAAATATTTTATGTTTTTTTTTTGAATAAAAATCTAGCTATTTAGTATAAGGGGAAAAAATATAACTCCAAATTAATATTAAAG	TTATCTATTGTTTTTCTAAGACGCATAAAATAAGCATGGATGCATGTAGTACACATACATATATATATTATCCAAAAAAATGTTTATAAATTTGTGCATT
			PBANKA_1416700	glycerol-3-phosphate 1-O-acyltransferase (G3PAT)	Unstudied	ACAGTGAACGGGCGTGACAG	ATATGGGTAGCACCAAGTGG	CGTATTTATACGATATATATATATGTTAACAAGTAATCATCTTGAATCGTGCCTTAAAGTTTGACCTATAACATTGTTAATATTGTGATTCCTAAAAATG	CTATTTTATCAATGTAATTGAATTTATTACTTTTATTAATTTCTTTCAATTTTATGAAGGGAAATGGACTATGACAAAAAATAATCATCCTATTTTTAAA
			PBANKA_0304000	pentafunctional AROM polypeptide, putative (AROM)	Unstudied	TTGCTGAACTGATACTAAGG	TTACGGGAAATATAGACAAA	AGTAATGTGTATCCATATATAAAATAATTTTTTTATTATAAAAATAAATAATTAAATTTAGTTAAATATTGTTATATACCTTTGATGCTTTATTTTAACT	TTATATTTTTTTATTTTTATGTGTTTTAAAAAATTATTTCAATCGATTTTAAATAAATCATTTTAAAAACAAAACAAATTGTTATACTAATAATAAATCA
			PBANKA_0306200	patatin-like phospholipase 1, putative (PATPL1)	Unstudied	ATTCTATCTCTTGACAGTGG	GTACGTACTCATATGGCTCT	TAAAGAAAAGCGTCTTTCTTTCCTTTCTTTATTATTATTTTTTTTTTTTTTACAAACTTTTTAATTTTTCACAATTTAATGATATAAAAAAAAAAAAAAAA	GTACATATATGCGTATGTGGCTATTAGTTTGTTTGCTTTTCTTTTTATTTGAAAGTAATATATAAAATGTGTACATATAAAGTATTTATTTTGGGTGTGA

			PBANKA_1360100	nucleoside transporter 1 (NT1)	Unstudied	CTAGTTGGTAACAACACTCT	AATCATCGCAAAAATCGGCA	ATATATTTCCCAAATATTATTTGAGAAAAAAATATAAACATTTTGCGAATTTTAATATATTTATTTAATTATTTTTTTCTATTTTTCCATAATAAGTCAAG	AAAAAACAATAAAGCATACAGTATAGCATACAATTGATGTATATTTATTTTTACATATGTGATCATATTATGTTTAAATAAATCAATGTGTGCCTCTAAT
			PBANKA_0824700	thioredoxin reductase, putative (TRXR)	Unstudied	TGAGTGACTTCACCTGCATT	CAAGGTGTAACATTTATGTG	AAACAAGCATACATTAATTTTTCTTTTTCAAACATGCCCTCATTTTTATGCACAAAATAGCTTTTTATTCGGATTAAAAAAAAAAAAAAAAAAAAAAAATT	ACAAAATTTGATATAAATAGGACCTATATATTTTTTCATAATTTATATATTATTATTCTATATTTTATTACATCTAAAATATCTTTAAAAAAGTGAAACA
			PBANKA_1127600	glucosamine 6-phosphate N-acetyltransferase, putative (GNA1)	Unstudied	TATGAATTCAGTTAGTAAGG	CTATCACAATCGTTTATTTC	ACATTTGCTTTTAATGAAATGTGATTTATATTTCTATATAGGTATATAATATTTTTTTCATACGGAATTTATATGTAAAAAGAAACTTAATTTTTAAAAA	TAAGAAAATACGAAAAAAAAATACATAAAATAATAAAAAAGGCACAAATAAATATTAATACAATATTTCGACATTTGGAAGCCCACAAAAAATATTTCTCT
			PBANKA_1027000	serine palmitoyltransferase, putative (SPT)	Unstudied	CATGGACCACGAATGTTAGG	AATTGGTATACAAACATCCG	GAATAAAAATAGAAAGCGTGATTTTTAATTCTAGATAAAAAATTAGATAAAGGATATCAAGAAGTAGGTAAATCACGATATAATACAAGAAATAGTAATA	TTATATTTTATATATTTTAGGATTGTATTAGAAGTAGAAAAAAATATATTAATACATGCTTGAATAAAAAACATATATATATATATAAGCATAATATAAA
			PBANKA_1028900	diphthamide biosynthesis protein 1, putative (DPH1)	Unstudied	TTAGGGGATGTTACATATGG	AATTGGCTTTAATTCTGTTG	ATATAGATTTATGGGAGAAAAAACATTCCCATAAAATAAACAAAATTGGAAAACGGCATTGAATAAATTTTGTTGCTCATAGTTTACCAGAAATTAAAAA	TGATTTATATGCGATAGAAATGGATAGAATGGCCCCCCAAAATATTATAGTTTATGTTTATTATATTTTTTTATTTTCATAAATACATATGCATATGTAA
			PBANKA_1207300	diphthine methyl ester synthase, putative (DPH5)	Unstudied	TATCATTGGACTAGGGTTAG	ACTAGGGTTAGGGGATGAAA	TAAATGAAAAAATAAGTGATTATATATTAATTTGCGCGTATAATCTCATACATTTTTATGAATATACTCAGACACTTATAAAATTTTCATATTTGAAAAC	TTTCCACATTCATGTGACTATTCTCAGTTTATATGCATATAAACTTTTTTCTCAAACTGTTTCTTCTTATATTTGAATAATCCCTTTTTTGTCTTAAATT
			PBANKA_0506700	citrate synthase, mitochondrial, putative (CS)	Unstudied	TGTGTATATCGACTGATTTG	ACACCTAATAATGTGATTGG	AATATTTGCAATTTAAAGGGAAATTATATATTAGCAGTGTAGATATATTAATCCGCGCGCAAAAAAAAAACGAAAGTGTAACACATAATTTTTTTGCAAAA	TTATATCAGAACTAATGGGAGAAAAAAAAACAATTTTTGATGTATATATGCATAGTTGGAAAAACGAGAAGGAATTTTTGTGAACATTTTTCTTCAATGC
			PBANKA_1112400	orotate phosphoribosyltransferase, putative (OPRT)	Unstudied	ATAGATGATGTATTTACTTG	TTATTTGGAGCATCGTATAA	AATATTTAAGAAAAACATATTTATATTGACAAAATTTTTTATTGTTTATTTCACAATTTGAGATTTTTTTTTTTGGCGATTGAAAAAACTTTGAAAAAA	TTTGGAAAAATTAAAATTAAAAAAAAAAAATAGAGATAAAATAATGGTGATTTTTTTTTCTTTTTTAATCTGAGTTCTGTATTTACTTTCATAAGTTTTT

Table S5: Parasitemia counts for Figures 1, 3 and S7

Figure 1Ai											
	P. yoelii One plasmid		P. yoelii PCR-fragment		P. yoelii no donor						
	Mouse 1	Mouse 2	Mouse 1	Mouse 2	Mouse 1	Mouse 2					
Day 4	0	0.08417508418	0	0	0	0					
Day 5	0.09505703422	0.1895734597	0.09216589862	0	0	0					
Day 6	0.4562043796	0.2949852507	0	0.09057971014	0	0					
Day 7	2.97219559	2.364864865	0.1748251748	0.09033423668	0	0					
Day 8	3.646833013	5.975103734	0.4389815628	0.2915451895	0	0					
Day 9	N/A	N/A	3.093721565	2.844036697	0	0					
Day 10	N/A	N/A	7.527881041	5.838041431	0	0					
Day 11	N/A	N/A	6.107566089	5.450941526	0	0					
	P. berghei One plasmid		P. berghei PCR-fragment		P. berghei no donor						
	Mouse 1	Mouse 2	Mouse 1	Mouse 2	Mouse 1	Mouse 2					
Day 4	0.09615384615	0.1953125	0.0946969697	0	0	0					
Day 5	0.4901960784	0.198019802	0.1968503937	0.09514747859	0	0					
Day 6	1.532912534	1.168014376	0.09250693802	0.09950248756	0	0					
Day 7	3.818827709	3.062200957	0.09174311927	0.1782531194	0	0					
Day 8	7.446808511	8.252427184	0.4	0.2906976744	0	0					
Day 9	7.632093933	4.812319538	1.263362488	0.8204193254	0	0					
Day 10	N/A	N/A	4.929577465	2.235179786	0	0					
Day 11	N/A	N/A	11.34020619	6.150793651	0	0					
**Figure 1Aii**											
	One plasmid, 500 bp		One plasmid, 250 bp		One plasmid, 100 bp		One plasmid, 50 bp				
	Mouse 1	Mouse 2	Mouse 1	Mouse 2	Mouse 1	Mouse 2	Mouse 1	Mouse 2			
Day 4	0	0	0	0	0	0	0	0			

Day 5	0.1742160279	0.2617801047	0.1813236627	0	0	0.0998003992	0	0			
Day 6	0.6711409396	1.391650099	0.1943634597	0.1872659176	0.3518029903	0.2961500494	0	0			
Day 7	4.12979351	4.8	0.826446281	0.8952551477	0.5703422053	1.157184185	0	0			
Day 8	6.343283582	6.048780488	5.135658915	6.978879706	3.796203796	3.237410072	0.580270793	0.3549245785			
Day 9	N/A	N/A	N/A	N/A	5.221674877	4.835589942	2.263779528	1.833976834			
Day 10	N/A	N/A	N/A	N/A	N/A	N/A	5.722326454	5.801376598			
	PCR-fragment, 500 bp		PCR-fragment, 250 bp		PCR-fragment, 100 bp		PCR-fragment, 50 bp				
	Mouse 1	Mouse 2	Mouse 1	Mouse 2	Mouse 1	Mouse 2	Mouse 1	Mouse 2			
Day 4	0	0	0	0	0	0	0	0			
Day 5	0	0	0	0	0	0	0	0			
Day 6	0.09910802775	0	0	0	0	0	0	0			
Day 7	0.1843317972	0.09009009009	0.1945525292	0.09115770283	0	0	0	0			
Day 8	0.9460737938	1.764705882	2.418604651	0.5623242737	0.6641366224	0.1976284585	0.7627118644	1.479289941			
Day 9	2.389705882	3.182256509	2.93040293	1.868327402	1.320754717	1.98019802	1.744186047	2.189781022			
Day 10	4.658077304	5.454545455	3.734827264	4.326923077	3.61328125	3.746397695	2.756653992	3.424657534			
**Figure 1C**											
	PbGIMO			FLAG-Cas9							
	Mouse 1	Mouse 2	Mouse 3	Mouse 1	Mouse 2	Mouse 3					
Day 1	0.08503401361	0.09310986965	0.2985074627	0.1683501684	0.08710801394	0.1718213058					
Day 2	0.3894839338	0.285986654	0.4212299916	0.5576208178	0.2879078695	0.1774622893					
Day 3	1.56114484	1.617507136	2.00729927	2.140945584	2.042801556	1.561065197					
Day 4	5.284147557	4.545454545	6.338028169	5.678537055	6.932573599	5.544747082					

Day 5	7.605633803	6.951871658	9.067357513	7.222222222	7.047619048	8.600182983					
**Figure 1F**											
	pPbU6-hdfr/yfcu Rap2/3-3xHA one plasmid		pPbU6-hdfr/yfcu Rap2/3-3xHA PCR-template		No donor						
Day 4	0	0	0	0	0	0					
Day 5	0.5115089514	0.2830188679	0.09803921569	0.1943634597	0	0					
Day 6	1.967799642	3.489531406	0.3787878788	0.5988023952	0	0					
Day 7	4.207699194	5.927342256	0.5870841487	0.6876227898	0	0					
Day 8	N/A	N/A	2.53411306	1.360544218	0	0					
Day 9	N/A	N/A	1.996007984	3.411306043	0	0					
Day 10	N/A	N/A	7.848837209	6.413994169	0	0					
**Figure 3Ai**											
	rap2/3 50 bp		rap2/3 100 bp		PBANKA_1224200 G1 50 bp		PBANKA_1224200 G1 100 bp				
	Mouse 1	Mouse 2	Mouse 1	Mouse 2	Mouse 1	Mouse 2	Mouse 1	Mouse 2			
Day 4	0	0	0,05	0	0	0	0	0			
Day 5	0,05	0	0,8	0,2	0	0	0,1	0			
Day 6	0,1	0	2,4	2,3	0	0,05	1,6	0,3			
Day 7	0,3	0,08	4,3	3,4	0	0,08	3,5	1,2			
Day 8	1,05	1,08	2,25	5,42	0,4	0,2	3,95	2,63			
Day 9	2,19	3,34	1,78	4,81	2	1,19	2,7	2,38			
Day 10	2,72	4,31	2,22	3,25	1,62	1,86	1,71	1,52			
**Figure 3Aii**											
	PBANKA_1224200		PBANKA_1225600		PBANKA_0914500		PBANKA_0622900		PBANKA_1451000		PBANKA_0829400
	G1	G2	G1	G2	G1	G2	G1	G2	G1	G2	G1
Day 4	0,4	0,3	0,3	0,3	0,2	0,1	0,2	0	0,3	0,1	0
Day 5	1,5	0,2	0,1	0,3	0,1	0,5	0,4	0,3	0,4	0	0
Day 6	5	1,3	0,4	2	0	2,5	2,5	2,5	4,4	0,3	0,2
Day 7		3,2	0,4		0,4			4,4		2	0,6
Day 8			2,3		0,8					4	1,3
Day 9					1,6						2,3

Day 10					3,6						4,6
**Figure S7A**											
	rap2/3 +gRNA		rap2/3 -gRNA		Mock (no DNA)						
	Mouse 1	Mouse 2	Mouse 1	Mouse 2	Mouse 1	Mouse 2					
Day 4	0.1	0.1	0	0	0	0					
Day 5	0.76	0.84	0	0	0	0					
Day 6	2.2	2.88	0	0	0	0					
Day 7	3	3.3	0	0	0	0					
Day 8	2.6	3	0	0	0	0					
Day 9	3.98	5.2	0	0	0	0					
Day 10	5.36	9.4	0	0	0	0					
**Figure S7B**											
	rap2/3 -gRNA		p25 KO								
	Mouse 1	Mouse 2	Mouse 1	Mouse 2							
Day 4	0	0	0	0							
Day 5	0	0	0	0							
Day 6	0	0	0	0							
Day 7	0	0	0.1	0.2							
Day 8	0	0	1	1.3							
Day 9	0	0	1.9	2.56							
Day 10	0	0	2.61	3.82							
Day 11	0	0	4.1	5.55							

Table S6: PbHiT classification of phenotype compared to *Plasmo*GEM and *piggyBac* screens

			**Disruptability**		
**Target gene ID**	**Gene name**	**PlasmoGEM phenotype**		**piggyBac phenotype**	**PbHiT phenotype**
PBANKA_1459900	signal recognition particle receptor subunit beta, putative (SRPRB)	Essential	Refractory	Possible	Not assigned
PBANKA_0804000	60S ribosomal protein L37, putative (RPL37)	Essential	Refractory	Refractory	Refractory
PBANKA_0809200	ribosomal protein L35, apicoplast, putative	Essential	Refractory	Refractory	Refractory
PBANKA_0703200	ribosomal protein L21, apicoplast, putative	Essential	Refractory	Refractory	Refractory
PBANKA_0505400	conserved Plasmodium protein, unknown function	Essential	Refractory	Refractory	Refractory

PBANKA_0812300	spindle and kinetochore-associated protein 1, putative (SKA1)	Essential	Refractory	Refractory	Not assigned
PBANKA_1009800	cytochrome c oxidase assembly protein COX15, putative (COX15)	Essential	Refractory	Refractory	Refractory
PBANKA_1136500	conserved Plasmodium protein, unknown function	Essential	Refractory	Refractory	Refractory
PBANKA_0914900	onserved protein, unknown function	Essential	Refractory	Refractory	Refractory
PBANKA_0817800	conserved Plasmodium protein, unknown function	Essential	Refractory	Possible	Refractory
PBANKA_1233900	50S ribosomal protein L14, mitochondrial, putative	Essential	Refractory	Refractory	Refractory
PBANKA_0514900	ookinete surface protein P28	Slow growers	Possible	Refractory	Not assigned
PBANKA_1362100	tyrosine kinase-like protein, putative (TKL3)	Slow growers	Possible	Possible	Not assigned
PBANKA_1108400	single-stranded DNA-binding protein, putative (SSB)	Slow growers	Possible	Refractory	Not assigned
PBANKA_1322400	exonuclease V, mitochondrial, putative	Slow growers	Possible	Refractory	Not assigned
PBANKA_1426400	mitochondrial carrier protein, putative	Slow growers	Possible	Possible	Not assigned
PBANKA_0314200	calcium-dependent protein kinase 1 (CDPK1)	Dispensable	Possible	Refractory	Not assigned
PBANKA_0616700	NIMA related kinase 4 (NEK4)	Dispensable	Possible	Refractory	Possible
PBANKA_0408200	calcium-dependent protein kinase 3 (CDPK3)	Dispensable	Possible	Possible	Possible
PBANKA_1305200	serine/threonine protein kinase, putative	Dispensable	Possible	Possible	Not assigned
PBANKA_1414500	glycogen synthase kinase-3 alpha, putative (GSK3alpha)	Dispensable	Possible	Refractory	Possible
PBANKA_0926600	protein GCN20, putative	Dispensable	Possible	Possible	Possible
PBANKA_1352600	serine/threonine protein kinase, putative	Dispensable	Possible	Refractory	Not assigned
PBANKA_1421600	calcium/calmodulin-dependent protein kinase, putative	Dispensable	Possible	Possible	Possible

PBANKA_0308500	tyrosine kinase-like protein, putative (TKL1)	Dispensable	Possible	Possible	Possible
PBANKA_0604400	targeted glyoxalase II, putative (tGLO2)	Dispensable	Possible	Possible	Possible
PBANKA_1013300	mitogen-activated protein kinase 1, MAPK1	Dispensable	Possible	Possible	Possible
PBANKA_1037800	secreted ookinete adhesive protein, SOAP	Dispensable	Possible	Possible	Possible
PBANKA_0515000	ookinete surface protein P25	Dispensable	Possible	Possible	Possible
PBANKA_1401600	methyltransferase, putative	Slow	Possible	Refractory	Not assigned
PBANKA_1034400	plasmepsin IV, PM IV	Slow	Possible	Not assigned	Not assigned
PBANKA_1104200	2-oxoisovalerate dehydrogenase subunit beta, mitochondrial, putative, BCKDHB	Slow	Possible	Possible	Not assigned
PBANKA_0103700	conserved Plasmodium protein, unknown function	unstudied		Refractory	Possible
PBANKA_0812900	conserved Plasmodium protein, unknown function	unstudied		Refractory	Possible
PBANKA_0603650	conserved Plasmodium protein, unknown function	unstudied		Not assigned	Possible
PBANKA_0409000	conserved Plasmodium protein, unknown function	unstudied		Refractory	Possible
PBANKA_0502900	conserved Plasmodium protein, unknown function	unstudied		Possible	Possible
PBANKA_0111400	conserved Plasmodium protein, unknown function	unstudied		Possible	Possible
PBANKA_0524100	conserved Plasmodium protein, unknown function	unstudied		Not assigned	Possible
PBANKA_0519200	conserved Plasmodium protein, unknown function	unstudied		Not assigned	Possible
PBANKA_0509600	conserved Plasmodium protein, unknown function	unstudied		Refractory	Possible
PBANKA_0315700	conserved Plasmodium protein, unknown function	unstudied		Possible	Possible
PBANKA_0519400	conserved Plasmodium protein, unknown function	unstudied		Not assigned	Possible
PBANKA_0519100	conserved Plasmodium protein, unknown function	unstudied		Not assigned	Possible

PBANKA_0821200	conserved Plasmodium protein, unknown function	unstudied	Refractory	Possible
PBANKA_0111500	conserved Plasmodium protein, unknown function	unstudied	Possible	Refractory
PBANKA_0817400	conserved Plasmodium protein, unknown function	unstudied	Refractory	Refractory
PBANKA_0408800	conserved Plasmodium protein, unknown function	unstudied	Refractory	Refractory
